# Biodegradable graphene nanocomposites as functional biomaterials: a review of their role in controlled drug delivery and tissue engineering

**DOI:** 10.1039/d5ra06280b

**Published:** 2025-11-19

**Authors:** Md. Mohiuddin, Md. Mahbubur Rahman, Md. Nizam Uddin, Rakib Hasan, Ismail Rahman

**Affiliations:** a Department of Mechanical Engineering, Khulna University of Engineering & Technology Khulna 9203 Bangladesh mahbub_rahman@me.kuet.ac.bd; b James C. Morriss Division of Engineering, Texas A&M University-Texarkana 7101 University Ave Texarkana TX 75503 USA muddin@tamut.edu; c Department of Mechanical, Aerospace, and Industrial Engineering, The University of Texas at San Antonio 1 UTSA Circle San Antonio TX 78249 USA; d Institute of Environmental Radioactivity, Fukushima University 1 Kanayagawa Fukushima City Fukushima 960-1296 Japan immrahman@ipc.fukushima-u.ac.jp

## Abstract

Biodegradable graphene nanocomposites (BGNs) have emerged as highly versatile platforms at the intersection of nanotechnology, materials science, and biomedicine. By combining the exceptional physicochemical properties of graphene-based materials with the biocompatibility and environmental sustainability of biodegradable polymers, BGNs constitute a unique class of materials for advanced biomedical applications. Key features of BGNs, such as high surface area, tunable surface chemistry, excellent mechanical strength, and the ability to interface effectively with biological systems, make them promising candidates for controlled drug delivery and tissue engineering. In drug delivery, BGNs facilitate high drug loading and enable spatially and temporally controlled release, which can be triggered by internal or external stimuli, thereby improving therapeutic efficiency while minimizing side effects. In tissue engineering, the mechanical robustness and customizable structure of BGNs support cellular attachment, proliferation, and differentiation, rendering them suitable as scaffolds for regenerating bone, cartilage, skin, and neural tissues. This review explores recent advancements in the fabrication techniques and biomedical applications of BGNs, emphasizing their role in achieving precise drug delivery and effective tissue regeneration.

## Abstract 2

Biodegradable graphene nanocomposites integrate graphenes' superior physicochemical properties of graphene with biodegradable polymers to enable advanced biomedical applications of stimuli-responsive drug delivery and tissue engineering scaffolds.

## Graphical abstract

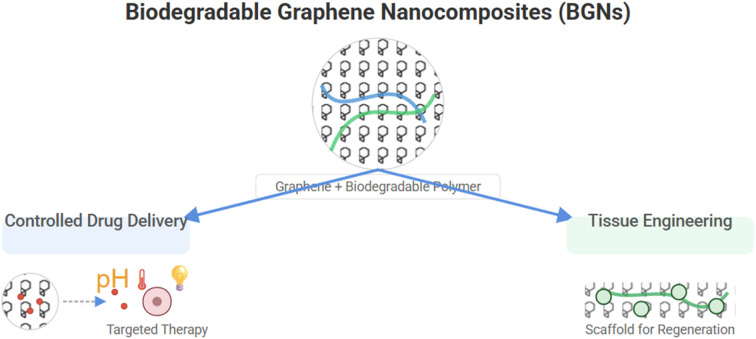

## 1 Introduction

### 1.1 Background and rationale

Conventional drug administration systems present several limitations, including poor bioavailability, uncontrolled release kinetics, off-target effects, and systemic toxicity.^1,2^ Similarly, traditional materials used in tissue repair often lack the requisite biocompatibility and mechanical properties, leading to failed integration with native tissue, implant failure, and adverse immune responses.^3^ These significant clinical challenges have motivated researchers to develop advanced materials capable of enhancing therapeutic efficacy, promoting predictable tissue regeneration, and minimizing adverse effects.

In recent decades, biomaterials science has progressed significantly, leading to the introduction of biodegradable polymers for biomedical applications. These polymers offer favorable properties, including minimal toxicity and high biocompatibility.^4^ In drug delivery, they enable stimuli-responsive (*e.g.*, to temperature, pH, light) controlled release of therapeutic agents,^5^ while in tissue engineering, they provide a temporary structural support that degrades in coordination with new tissue formation.^6^ However, the utility of many biodegradable polymers is constrained by inherent limitations in mechanical strength, electrical conductivity, or targeted functionality, properties which are essential for more demanding applications like load-bearing tissue repair or electro-active tissue stimulation.^4,7^

To overcome these shortcomings, research has shifted toward nanotechnology, which enables the engineering of materials at the molecular scale. Among various nanomaterials, graphene and its derivatives, graphene oxide (GO) and reduced graphene oxide (rGO), have gained substantial attention. Their exceptional characteristics, including an extremely high surface-area-to-volume ratio, low weight, excellent thermal and mechanical properties, and the capacity to adsorb drugs or bioactive molecules *via* non-covalent interactions, make them ideal reinforcing and functional agents.^8^ However, the standalone use of pristine graphene-based materials faces its own challenges, including potential cytotoxicity,^9^ poor physiological stability, and a tendency to aggregate in aqueous biological environments, which can limit their practical application.^10^

Consequently, a synergistic approach has emerged: incorporating these nanomaterials into biocompatible polymeric matrices to improve their biostability, biocompatibility, and overall functionality. This has led to the development of BGNs, a class of materials that strategically combines the functional advantages of graphene with the safety and biodegradability of polymers. In these composites, graphene provides significant mechanical reinforcement, high drug-loading capacity, and responsiveness to external stimuli. Simultaneously, the polymer matrix ensures biocompatibility and produces safe, absorbable degradation byproducts. Furthermore, BGNs are valued for their sustainable nature, especially when fabricated with natural polymers, addressing the growing need for environmentally conscious medical technologies.

### 1.2 Scope and methodology

Numerous reviews have addressed the biomedical applications of biodegradable polymers and graphene-based materials separately. Comprehensive analyses exist for chitosan-functionalized GO in cancer therapy,^11^ GO-based hydrogels for drug delivery,^12^ natural polymeric nanobiocomposites for anti-cancer therapeutics,^13^ and polysaccharide-based nanomedicines for cancer immunotherapy.^14^ Other reviews have covered topics such as plasma modification of drug delivery systems,^15^ bacterial cellulose for wound dressings,^16^ 3D bioprinting with chitosan,^17^ and nanostructured composites for bone regeneration.^18^

**Fig. 1:**
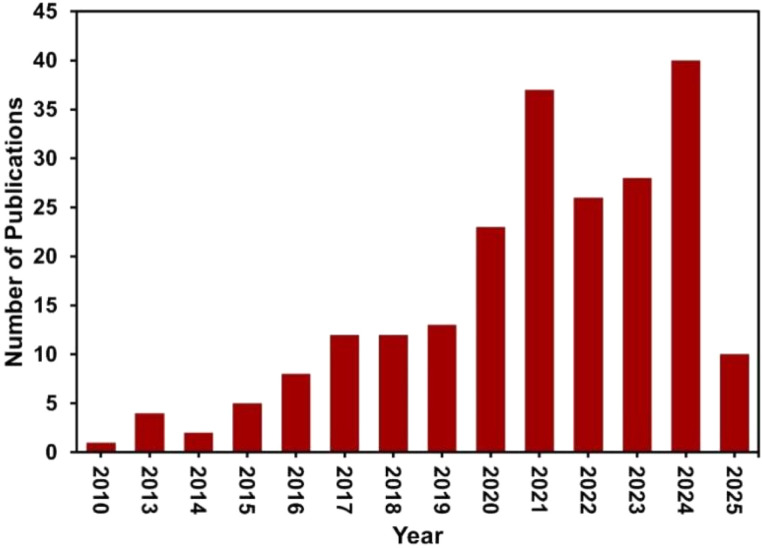
Annual number of publications on BGNs since 2010. Data sourced from ‘https://scopus.com’.

Although these articles provide valuable insights, most focus on either drug delivery or tissue engineering, and many concentrate solely on either biodegradable polymers or graphene. Reviews that explore the synergy of polymer–graphene nanocomposites in both drug delivery and tissue engineering are limited. This highlights a gap in the literature: a comprehensive review that simultaneously addresses both applications using BGNs and explores advancements in their fabrication is lacking. As shown in Fig. 1, research on BGNs for drug delivery and tissue engineering has gained significant attention since 2014, indicating a rapidly emerging field that warrants an in-depth, integrated review.

**Fig. 2:**
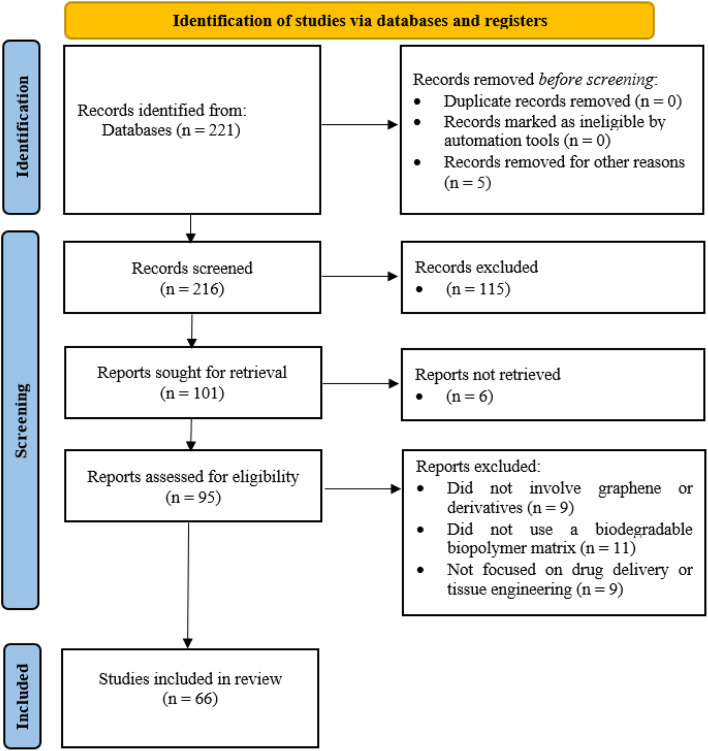
PRISMA flow diagram illustrating the study selection process for the systematic review.

This review bridges this gap by systematically examining both the fabrication techniques and the dual applications of BGNs in controlled drug delivery and tissue engineering. A comprehensive literature search was conducted using the Scopus database with the keywords: *(“biopolymer” OR “natural polymer” OR “biodegradable polymer”) AND (“graphene” OR “graphene oxide” OR “rGO”) AND (“nanocomposite” OR “nanomaterial”) AND (“drug delivery” OR “controlled release”) OR (“tissue engineering” OR “regenerative medicine”)*. This query yielded 221 records published between 2010 and June 2025. Following the PRISMA guidelines (Fig. 2), records were screened, and 66 primary research articles meeting all inclusion criteria were selected for synthesis. These core studies, supplemented by additional references for background context, form the basis of this review.

## 2 Foundational materials

### 2.1 Graphene and its oxidized derivatives

**Fig. 3:**
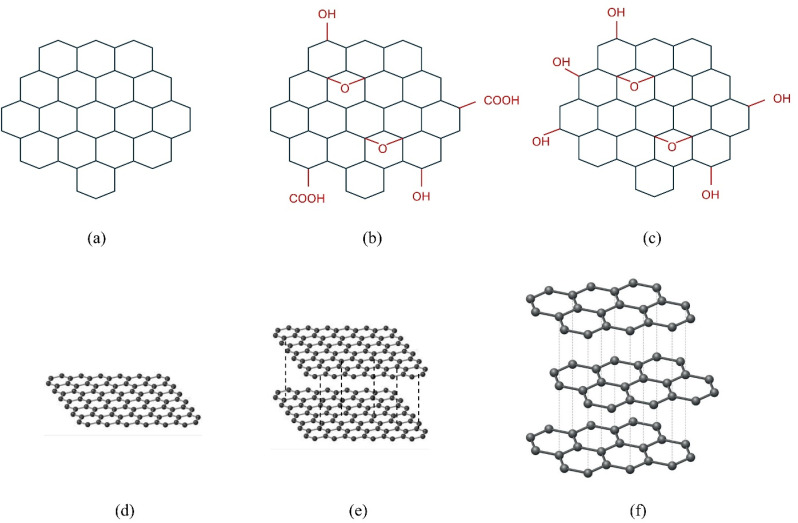
Schematic representations of (a) pristine graphene, (b) graphene oxide, (c) reduced graphene oxide, and 3D models of (d) single-layer graphene, (e) bi-layer graphene, and (f) tri-layer graphene stack.

Graphene is a single layer of carbon atoms arranged in a two-dimensional honeycomb lattice (Fig. 3a). Each carbon atom is sp^2^ hybridized, forming strong covalent bonds approximately 0.142 nm in length.^19^ This unique structure imparts exceptionally high electrical conductivity, mechanical strength (with a Young's modulus of ∼1 TPa), thermal conductivity, and a large theoretical specific surface area (∼2630 m^2^ g^−1^). Each carbon atom in graphene is covalently bonded to three neighboring atoms, forming a robust and stable hexagonal framework. This sp^2^ bonding provides delocalized π-electrons across the basal plane, which are responsible for graphene's high conductivity and chemical inertness.^20^ While chemically inert, its surface can be functionalized through covalent or non-covalent methods to attach biomolecules or drugs, though its intrinsic hydrophobicity can be a challenge for biological dispersion.^21,22^ Such functionalization exploits graphene's π-system for noncovalent π–π stacking with aromatic drug molecules, while covalent grafting (*e.g.*, carbodiimide-mediated amidation) allows linkage of –COOH groups on GO with –NH_2_ groups of polymers like chitosan. Similarly, hydroxyl and epoxy groups on GO readily form hydrogen bonds with hydrophilic polymers (*e.g.*, PVA, cellulose), enabling stable polymer–graphene nanocomposites. These interfacial interactions dictate dispersion, mechanical reinforcement, and drug release behavior. Such functionalization strategies are crucial for biomedical applications because they allow the attachment of drugs, peptides, or targeting ligands, improving dispersibility and specificity.^23^ Common synthesis methods include mechanical exfoliation, which yields high-quality flakes but lacks scalability, and chemical vapor deposition, which allows for large-area film production.^24^ Other routes include epitaxial growth on SiC substrates,^25^ unzipping of carbon nanotubes,^26^ and chemical reduction of GO.^27^ Each method produces graphene with different levels of purity, layer control, and defect density, which in turn affect its chemical reactivity and biomedical performance.

GO is derived from graphite *via* aggressive oxidation and subsequent exfoliation. Its structure contains a high density of oxygen-containing functional groups (hydroxyl, epoxy, and carboxyl groups) that disrupt the planar sp^2^ network, introducing sp^3^-hybridized carbon atoms and rendering it electrically insulating but highly hydrophilic and easily dispersible in water (Fig. 3b).^22^ This aqueous dispersibility and the abundance of functional groups for further chemical modification make GO particularly attractive for biomedical applications such as drug delivery, biosensing, and tissue engineering.^28^ The type and density of oxygen functionalities depend on the oxidation method used (Hummers', Brodie's, or Staudenmaier's), which not only influence dispersibility but also toxicity and stability.^29,30^ Hummers' method involves strong acids (typically sulfuric acid) and oxidants like potassium permanganate to rapidly oxidize graphite, producing GO with various oxygen functional groups.^31^ Brodie's method, one of the earliest, uses fuming nitric acid and potassium chlorate, resulting in slower oxidation and more defects.^32^ Staudenmaier's method improves on Brodie's by combining concentrated sulfuric and nitric acids with potassium chlorate for faster oxidation and higher oxygen content, but it produces hazardous chlorine dioxide gas.^33^ These oxygen functionalities (–OH, –COOH, –O–) provide chemical handles for bonding with polymers: –COOH groups undergo esterification or amidation with polymeric hydroxyl/amine groups, while hydroxyl and epoxy moieties participate in hydrogen bonding and ionic crosslinking. Such interactions govern composite stability, swelling, and enzymatic degradation in physiological environments.

rGO is produced by removing a significant portion of the oxygen-containing groups from GO through chemical, thermal, or electrochemical reduction (Fig. 3c). This process partially restores the sp^2^ carbon network, improving electrical conductivity to a level between that of pristine graphene and GO, although residual defects remain.^19,34^ Reduction routes include chemical reductants (*e.g.*, hydrazine, sodium borohydride, or green alternatives such as ascorbic acid), high-temperature annealing, and electrochemical reduction. Each pathway affects the residual oxygen content and defect density, which determine conductivity, dispersibility, and biocompatibility.^35^ Chemical reduction of GO typically involves the use of strong reducing agents like hydrazine or sodium borohydride, which react with oxygen-containing functional groups (such as epoxides, hydroxyls, and carboxyls) to remove them from the GO surface. This significantly restores the conjugated sp^2^ carbon network but can introduce defects and leave toxic residues, negatively affecting electrical and structural properties.^35^ Green reductants like ascorbic acid operate through similar mechanisms but involve milder redox reactions, offering moderate deoxygenation while maintaining better biocompatibility and aqueous dispersibility.^36^ Thermal annealing, on the other hand, works by heating GO to high temperatures (typically 500–1100 °C) under inert or reducing atmospheres (*e.g.*, argon or hydrogen), which causes decomposition of oxygen functional groups and reconstructs sp^2^ domains. This method yields highly conductive rGO but often at the expense of scalability and may cause layer restacking.^37^ Finally, electrochemical reduction involves applying a potential across GO films in an electrolyte solution, triggering electron transfer that selectively removes oxygen groups. This technique avoids harsh chemicals and allows fine-tuning of reduction levels, producing rGO with controlled surface chemistry and good dispersibility.^38^ The residual oxygen groups on rGO still permit limited hydrogen bonding or ionic interactions with polymers, while defect sites act as nucleation points for covalent grafting. Thus, tuning the reduction level enables control over degradation rate, electrical conductivity, and mechanical reinforcement of the resulting nanocomposites. The resulting material offers a tunable balance of conductivity and dispersibility, making rGO a cost-effective and versatile option for similar biomedical applications.^19,24^

Graphene materials can exist as monolayer, bilayer, trilayer, or multilayer sheets (Fig. 3d–f). As the number of layers increases, the specific surface area and some unique quantum electronic properties decrease, but the potential for functionalization for biomedical use remains a key feature.^22,24^ Specifically, one, two, and three layers are termed monolayer, bilayer, and trilayer graphene, respectively, while 5–10 layers are considered few-layer graphene and 20–30 layers as multilayer graphene (nanocrystalline thin graphite).

### 2.2 Common biodegradable polymers

Biodegradable polymers, derived from both natural and synthetic sources, are central to the development of BGNs. They provide essential biocompatibility and degrade into non-toxic byproducts that can be safely metabolized or excreted by the body, aligning with the need for sustainable and eco-friendly biomedical solutions.^39^ When combined with graphene, these polymers form composites with enhanced mechanical strength, electrical conductivity, and tailored functionality, making them ideal for drug delivery and tissue engineering.^40^

Table 1 provides an overview of common biodegradable polymers used in BGNs, detailing their origin, formulation possibilities, and key functional properties.

**Table 1:** Overview of biodegradable polymers for drug delivery and tissue engineering applications

Polymer	Origin/type	Formulation possibilities	Key functional properties	References
**(A) Natural proteins**				
Silk fibroin (SF)	Silkworm-derived (*Bombyx mori*)	Fabricated as hydrogels, porous sponges/scaffolds, films, fibers, and nanoparticles	High mechanical strength; tunable degradation; minimal immunogenicity	41 and 42
Gelatin	Denatured collagen from animal bone/skin	Used in hydrogels, porous scaffolds, films, microspheres, and electrospun nanofibers	Non-toxic and non-immunogenic; promotes cell adhesion (collagen-mimetic RGD sequences)	43 and 44
Collagen (COL)	Major ECM protein from connective tissues	Sponges/fibrous scaffolds, hydrogels, sheets (*via* molding, electrospinning, 3D bioprinting)	Low immunogenicity; contains natural cell-binding sites (RGD) and directs cell behavior	45
Zein	Corn protein (GRAS excipient)	Films/coatings, nanoparticles, electrospun fibers, scaffolds	Biodegradable, biocompatible protein; hydrophobic (alcohol-soluble) enabling controlled drug release; good film-former with moderate mechanical strength; supports drug-loaded membranes and tissue scaffolds	46

**(B) Natural polysaccharides and derivatives**				
Chitosan (CS)	Derived from chitin in crustacean shells	Processed into hydrogels, nanoparticles, nanofibers, membranes, films, and 3D scaffolds	Inherently mucoadhesive and hemostatic; antimicrobial; low immune rejection	47–49
Alginate	From brown seaweed	Formed into ionically crosslinked hydrogels/gels, porous foams/sponges, microcapsules/microspheres, fibers, and films	Low-toxicity; undergoes mild Ca^2+^-induced gelation; yields high-porosity, tunable-stiffness networks	50
Cellulose nanofibers	Plant-derived nanoscale fibers	Fabricated into ionically crosslinked hydrogels, freeze-dried porous scaffolds/aerogels and composite matrices	Extremely high surface area and tensile strength; forms interconnected porous networks that support cell growth and may confer antibacterial effects	51
Starch	Plant polysaccharide (amylose/amylopectin)	Hydrogels (drug-loaded matrices), electrospun nanofibrous scaffolds, films, microparticles	Highly hydrophilic (water-absorbing); low-cost; promotes cell proliferation and wound healing	52 and 53
Agarose	Red algae-derived galactose polymer	Thermoresponsive hydrogels (injectable gels, bioinks), cryogels, sponges/scaffolds (*e.g.* 3D-printed)	Reversible gelation (thermo-sensitive sol–gel transition); good mechanical strength and high water retention; inert	54 and 55
Carboxymethyl cellulose (CMC)	Cellulose-derived anionic polysaccharide	Hydrogels, films, 3D porous scaffolds (*e.g.* for bone or soft tissue), wound dressings	Very hydrophilic (forms swellable gels); thixotropic (viscoelastic) with high viscosity	56
Hyaluronic acid	ECM glycosaminoglycan	Hydrogels (injectable gels, bioinks, cryogels), 3D-printed scaffolds, composite matrices; also viscous solutions/films (*e.g.* eye drops, dermal fillers)	Mucoadhesive (interacts with tissues like cartilage, skin); highly hydrophilic (excellent water retention); tunable viscoelasticity and porosity	57
Arabinoxylan (ARX)	Plant hemicellulose	Hydrogel matrices (films, injectable gels, tablets, capsules) and particulate systems (micro/nanogels)	Polysaccharide; forms gel networks for controlled release; shown to promote wound healing	58 and 59
β-Glucan (BG)	Found in cereal grains, fungi, yeast cell walls	Porous scaffolds (*e.g.* freeze-dried foams, nanocomposites with hydroxyapatite) and hydrogels	Highly hydrophilic (water-adsorbing) polymer; supports cell attachment and proliferation	60
Guar gum (GG)	From guar bean (*Cyamopsis tetragonolobus*)	Injectable hydrogels, films/membranes, and freeze-dried scaffolds (often blended with other polymers)	Mucoadhesive polymer; exhibits gel-forming ability, high swellability and controlled-release characteristics	61 and 62
Chondroitin sulfate (CS-MA)	Cartilage ECM-derived sulfated polysaccharide	Photocrosslinked hydrogels (often blended with HA) for cartilage TE	Cartilage-mimetic biopolymer; provides hydration and growth-factor binding; tunable mechanics, swelling, and enzymatic degradability	63
Carboxymethyl arabinoxylan (CMARX)	Psyllium-derived hemicellulose	Hydrogels, polyelectrolyte nanoparticles (*e.g.* with CS), films, composite scaffolds	Biocompatible, biodegradable polysaccharide; CM modification increases crystallinity and thermal stability; supports pH-responsive swelling and sustained drug release	64
Kappa-carrageenan (K-CG)	Red algae-derived sulfated polysaccharide	Ionic hydrogels (beads, gels, membranes), composite scaffolds, electrospun mats	Forms strong ionically crosslinked gels (with K^+^/Ca^2+^); highly hydrophilic (swelling); biodegradable; excellent encapsulation and sustained release of growth factors/drugs	65
2-Hydroxyethyl cellulose (2-HEC)	Cellulose ether	Cryogels, hydrogels, films, microparticles	Biocompatible and biodegradable; highly hydrophilic with large swelling; forms viscous, stable gels; demonstrated use in sustained-release cryogel systems	66
Konjac glucomannan (KGM)	From konjac tuber	Hydrogels (thermal or alkali-induced gelation), sponges/aerogels, films	Biocompatible and biodegradable; extremely high viscosity and water uptake; strong gelation (acetyl-dependent); forms robust matrices for sustained drug release and wound dressings	67

**(C) Synthetic polyesters and copolymers**				
Poly(glycerol sebacate) (PGS)	Synthetic thermoset elastomer	Made into porous elastomeric scaffolds (foams), fibrous meshes, and composite grafts	Elastomer; rubber-like elasticity; tunable mechanical properties and degradation rate (matched to native soft tissues)	68 and 69
Poly(3-hydroxybutyrate) P(3HB)	Bacterial polyester	Scaffolds (*e.g.* bone, tissue engineering), cardiovascular patches, biodegradable microspheres/carriers	Intrinsically osteoinductive (promotes MSC osteogenic differentiation and bone regeneration)	70 and 71
Poly(ε-caprolactone) (PCL)	Aliphatic polyester	Processed into porous scaffolds (*e.g.* bone or vascular), electrospun fiber mats, and biodegradable microspheres/nanoparticles	Thermoplastic; good mechanical strength and toughness; easily fabricated (melt, 3D-printing, electrospinning) for sustained-release applications	72
Poly-l-lactic acid (PLLA)	Aliphatic polyester	3D porous scaffolds (electrospun fibers, meshes, foams), microspheres/nanoparticles (drug carriers), composites (*e.g.* with bioceramics for bone TE)	High tensile strength; tunable mechanical stiffness and degradation rate (*via* crystallinity/porosity); FDA-approved for implants	73 and 74
Polylactic acid (PLA)	Aliphatic polyester	3D-printed scaffolds, electrospun mats, films, microparticles, sutures	Biodegradable (hydrolyzes to lactic acid); high tensile strength and stiffness (brittle); hydrophobic; tunable crystallinity; biocompatible but relatively inert (often blended or surface-modified for cell adhesion)	75
Poly(lactic-*co*-glycolic acid) (PLGA)	Copolymer	Microparticles, nanoparticles, 3D scaffolds, implants	Biocompatible and biodegradable with tunable degradation rate (by LA/GA ratio); relatively hydrophobic; good mechanical strength; extensively used for controlled release of drugs, proteins and growth factors	76
Poly(propylene fumarate) (PPF)	Unsaturated polyester	Injectable/crosslinked hydrogels, 3D-printed scaffolds, composites	Biodegradable (degrades to fumaric acid and propylene glycol); tunable mechanical properties and degradation rate; inherently crosslinkable (unsaturated sites) for high-strength bone/regenerative scaffolds	77
Poly(glycolic acid-*co*-propylene fumarate) (PGA-*co*-PF)	Copolymer	Electrospun nanofibrous scaffolds, composite fibers	Biodegradable copolymer; high water uptake and accelerated degradation when formulated with nanofillers; reinforced scaffolds exhibit enhanced mechanical strength and osteoconductivity (*e.g.* with GO/hydroxyapatite)	78

**(D) Semi-synthetic methacrylated polymers**				
AESO (acrylated epoxidized soybean oil)	Acrylated vegetable oil	Photocurable 3D-printed resins and hydrogels (often composite with nano-hydroxyapatite for bone TE)	Thermoset resin; UV-crosslinkable (photopolymerizable) with tunable mechanics; supports cell growth/differentiation	79 and 80
Alginate-methacrylate (AlgMA)	Methacrylated alginate	Photocrosslinked hydrogels and 3D-printed scaffolds	Tunable stiffness and degradation (hydrolytic/enzymatic)	80
Gelatin-methacryloyl (GelMA)	Methacrylated gelatin	Photocurable hydrogels and bioinks for 3D bioprinting; microcarriers	Retains natural RGD and matrix metalloproteinases-cleavage sequences	81
Chondroitin sulfate-methacrylate (CS-MA)	Methacrylated cartilage ECM	Photocrosslinked hydrogels (often blended with HA) for cartilage TE	Cartilage-mimetic biopolymer; provides hydration and growth-factor binding; tunable mechanics, swelling, and enzymatic degradability	63

**(E) Natural or bioactive polymers**				
Peptides	Natural or synthetic short proteins/polypeptides	Self-assembling hydrogels and nanofiber networks (3D scaffolds), injectable gels, composite coatings; can form nanoparticles for drug delivery	Often bioactive (*e.g.* signaling peptides); good mechanical stability and tissue-like elasticity in hydrogel form; tunable assembly (*e.g.* β-sheet fibers)	82

### 2.3 Polymer–graphene interfacial interactions

The interactions at the polymer–graphene interface are a key determinant of the performance of BGNs, and they have a significant impact on important parameters like dispersion, mechanical augmentation, degradation, and drug release behaviors. Usually, these interfacial interactions are divided into two categories: covalent bonds which involve the sharing of electron pairs between atoms and result in strong, stable chemical linkages such as amide, ester, or radical-mediated grafts; and non-covalent interactions which are weaker and reversible forces that do not involve sharing of electrons such as hydrogen bonding, electrostatic forces, π–π stacking, and hydrophobic interactions. Different classes of biodegradable polymers tend to favor different types of bonding depending on their functional groups.

Hydrogen bonding is a specific type of non-covalent interaction that occurs when a hydrogen atom, covalently attached to an electronegative atom (such as oxygen or nitrogen) within a molecule, experiences an attractive force to another electronegative atom with lone electron pairs. In GO, the abundant oxygenated functional groups including hydroxyl (–OH), carboxyl (–COOH), and epoxy (–C–O–C) act as both hydrogen bond donors and acceptors, allowing physiochemical interactions with complementary sites on surrounding polymers.^83^ Natural polysaccharides and their derivatives (such as alginate, cellulose, starch, hyaluronic acid, and carboxymethyl cellulose), as well as natural proteins (collagen, gelatin, silk fibroin), are especially suited for forming hydrogen bonds with GO. Their molecular structures are rich in hydroxyl, carboxyl, and amide groups, providing ample sites for hydrogen bonding interactions with the oxygenated functionalities on GO.^83^ It enhances water dispersibility by stabilizing the nanocomposite network, boosts swelling by increasing the matrix's hydrophilicity, and enables better control of drug release by slowing burst release and supporting sustained release. A dense hydrogen-bonding network also strengthens mechanical properties and elasticity while regulating degradation, which is important for biomedical hydrogels and drug delivery systems.^84^

Electrostatic interactions are fundamental to the interfacial binding between biodegradable polymers and graphene derivatives. Specifically, positively charged groups on cationic biodegradable polymers such as the amine groups found in chitosan and polycationic peptides are attracted to the negatively charged carboxyl groups on GO or other oxidized graphene surfaces. This electrostatic attraction enhances compatibility and mechanical integrity in composite materials, which is especially important in biomedical applications that require controlled interactions and stable matrices.^85^ Similarly, anionic biodegradable polymers including alginate, hyaluronic acid, and CMC can interact with amine functionalized or protonated graphene derivatives through complementary charge pairing. The carboxyl and sulfate groups in these polysaccharides form strong ionic bonds with positively charged sites on modified graphene. These electrostatic interactions often lead to pH responsive behavior, as the ionization states of both polymer and graphene surfaces change with pH, enabling tunable adhesion and controlled drug release.^86,87^

The basal plane of graphene and rGO provides an extended π-conjugated system, which facilitates π–π stacking and hydrophobic interactions with biodegradable polymers containing aromatic rings or hydrophobic backbones. Proteins such as gelatin and peptides enriched with aromatic residues such as phenylalanine, tyrosine, and tryptophan offer abundant sites for π–π interactions, while biodegradable polyesters like PLA, PLLA, PCL, and PLGA possess hydrophobic domains that participate in both π–π stacking and hydrophobic contacts with graphene surfaces. These interactions enhance compatibility and matrix integrity by stabilizing polymer chains along the graphene plane, promoting uniform dispersion and superior reinforcement.^40,88^ π–π stacking is particularly efficient at adsorbing aromatic drug molecules, increasing entrapment efficiency and allowing controlled release profiles due to strong molecular interactions at the interface. Furthermore, hydrophobic contacts between graphene and biodegradable polymer chains reduce water uptake and permeability, improving barrier properties and retarding degradation rates under aqueous conditions, an effect desirable for prolonged drug delivery and enhanced packaging performance.^88^

Amide bond formation typically utilizes the carboxyl (–COOH) groups abundant on GO surfaces. These can be activated using carbodiimide chemistry, most commonly with EDC (*N*-ethyl-*N*′-(3-dimethylaminopropyl)carbodiimide) and NHS (*N*-hydroxysuccinimide), to form amide bonds with amine containing biopolymers such as chitosan, collagen, or gelatin. This reaction proceeds by forming an active ester intermediate on GO that readily reacts with amino groups on the polymer, resulting in a stable amide linkage that covalently anchors the polymer chain to the graphene surface.^89^

Esterification reactions exploit the reaction between GO's carboxyl groups and hydroxyl (–OH) functional groups present in hydroxyl-rich biopolymers, such as polysaccharides and polyesters. This process, often catalyzed by acid or activation agents, leads to the formation of ester bonds, chemically grafting the polymer backbone onto the graphene architecture and yielding a network with improved mechanical and degradation resistance.^89^

Radical grafting is widely used for methacrylated biopolymers like GelMA, AlgMA, or CS-MA. Under UV irradiation, these methacryloyl groups form free radicals that can react with activated double bonds on the GO surface or pre-functionalized graphene, leading to the formation of robust covalent crosslinks. This method is especially effective in hydrogel synthesis, resulting in nanocomposite networks with high crosslinking density and mechanical stability suitable for scaffolds.^90,91^

## 3 Fabrication of BGNs

The fabrication of BGNs involves a variety of techniques aimed at achieving optimal dispersion of graphene, mechanical integrity, and functional performance. The choice of methods depends heavily on the desired final form (*e.g.*, film, hydrogel, fiber, 3D scaffold) and application. Many studies employ hybrid approaches, combining multiple methods to leverage their respective advantages. For instance, sonication is often integrated with solvent casting or crosslinking to enhance nanoparticle dispersion. A comparative summary of the techniques discussed is provided in Table 2.

**Table 2:** Fabrication techniques for graphene–polymer composites: a comparative overview

Method	Process steps	Advantage/limitations	References
Solvent casting	Dissolve polymer and graphene (*e.g.* GO) in solvent; cast into mold; evaporate solvent to form film	Simple, cheap, and scalable yields uniform thin films; enhances mechanical, barrier, and antibacterial properties	92–94
		Limited 3D architecture control, potential nanoparticle aggregation, solvent residues	
Solution casting with sonication	As above but apply ultrasonic waves during mixing to disperse fillers; then cast and dry	Improved nanoparticle dispersion compared to plain casting; more uniform composites	92, 95 and 96
		Excessive sonication may degrade polymers, some aggregation and solvent residues may persist	
Emulsification and crosslinking	Create a (water-in-oil or double) emulsion of polymer solution containing graphene; solidify droplets by chemical crosslinking (*e.g.* GLA)	Enables formation of micro/nanoparticles with high surface area and uniform graphene dispersion for drug delivery; crosslinking provides stability and tunable release	83 and 97
		Use of surfactants/oil phases may affect biocompatibility; batch variability possible	
Sonication-assisted (hybrid) synthesis	Use ultrasonication to drive *in situ* reactions or mixing (*e.g.* solvothermal or co-precipitation) of graphene and other components	Enables efficient exfoliation and uniform dispersion of graphene, scalable and simple, can be combined with hybrid synthesis for multifunctionality	98 and 99
		Excessive sonication can cause defects and reduce flake size; risk of agglomeration or residual surfactants if not optimized	
*In situ* polymerization	Polymerize monomers in presence of graphene fillers (*e.g.* Michael addition, free-radical) to form network	Enables uniform graphene dispersion and strong interfacial bonding, leading to enhanced mechanical, thermal, and drug release properties; allows covalent grafting and functionalization	99 and 100
		Complex chemistry, need for initiators/catalysts, risk of aggregation at high graphene content, and potential cytotoxicity from residual chemicals	
Free-radical polymerization (lyophilization)	Initiate polymerization (*e.g.* acrylic monomers) in solution; freeze-dry the gel to form porous scaffold	Creates porous 3D scaffolds after freeze-drying; controllable pore structure	96 and 101
		Requires initiators and freeze-drying, which may affect drug stability and loading	
Magnetic functionalization (co-precipitation)	Synthesize magnetic nanoparticles (*e.g.* Fe_3_O_4_) onto graphene (GO) by co-precipitation; incorporate into polymer (*e.g. via* emulsification or casting)	Enables magnetically targeted drug delivery and imaging; stable integration of Fe_3_O_4_ enhances multifunctionality	102 and 103
		Potential agglomeration, requires precise chemical control	
Electrospinning	Apply high-voltage to polymer/graphene solution to draw nanofibers onto a collector; then (optionally) crosslink fibers (*e.g.* heat or ionic)	Produces nanofibrous mats that mimic ECM, supporting cell growth and sustained drug release	104 and 105
		Limited to thin mats and challenges in loading cells or drugs uniformly in 3D structures	
UV-assisted crosslinking	Mix photopolymerizable polymers with graphene and a photoinitiator; expose to UV light to induce covalent crosslinking into a network	Rapid solidification; spatial control of crosslinking; GO reinforcement enhances stiffness	106
		UV penetration depth limits thickness; photoinitiators may introduce toxicity	
3D printing (additive manufacturing)	Extrude thermoplastic melt or hydrogel ink layer-by-layer to build 3D scaffold; may include post-print coatings	Enables precise, patient-specific scaffold geometry. Surface coatings (*e.g.* polydopamine) can aid GO deposition	107 and 108
		High processing temperatures may limit cell/drug loading and can affect sensitive biomolecules	

### 3.1 Solution-based and casting methods

#### 3.1.1 Solvent casting

**Fig. 4:**
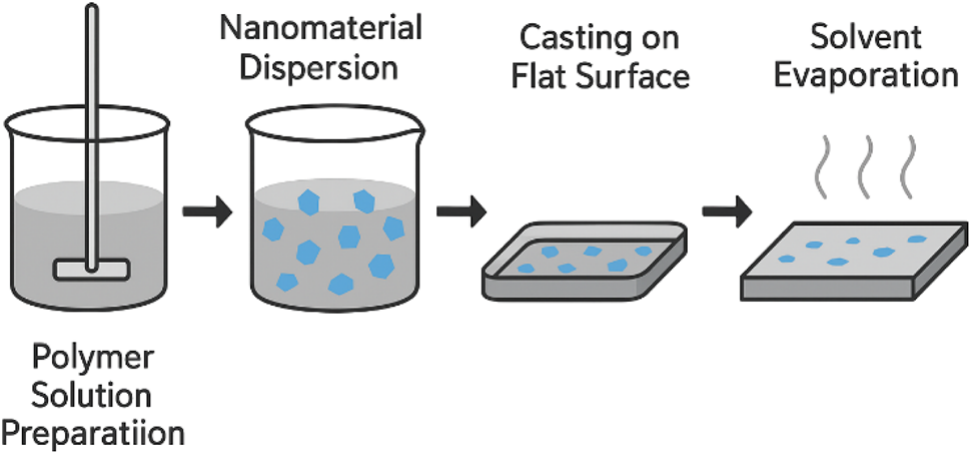
Schematic representation of the solvent casting process for nanocomposite thin films.

This widely used and straightforward method involves dissolving a polymer and dispersing graphene in a suitable solvent, casting the mixture onto a flat surface, and slowly evaporating the solvent to form a solid film (Fig. 4).^109–111^ During this process, solvent molecules act as mediators that control both polymer chain mobility and graphene dispersion within the solution. The viscosity of the casting solution and the polarity of the solvent influence the degree of mixing, with well-matched solvents promoting uniform dispersion and poor solvents increasing the risk of aggregation.^112^ As the solvent evaporates, the polymer chains reorganize and gradually entrap graphene sheets, locking in the noncovalent interactions (hydrogen bonding, electrostatic forces, or π–π stacking) formed in solution. The evaporation rate is a critical factor: slow solvent removal facilitates homogeneous film formation, whereas rapid evaporation can lead to nanoparticle migration or surface aggregation. In some systems, residual oxygen-containing groups on graphene derivatives may undergo limited condensation reactions with polymer hydroxyl or amine groups during drying, forming ester or amide linkages that further stabilize the composite structure.^113^ Solvent casting remains the simplest fabrication route, but its lack of 3D control makes it unsuitable for regenerative scaffolds compared to electrospinning or 3D printing. For instance, SF/GO nanocomposite films were fabricated by blending the components in solution, where the presence of GO induced a conformational transition in the SF matrix from a random coil to a more stable β-sheet structure, thereby enhancing mechanical integrity.^114^ This technique has also been used to create 2-HEC/graphene films with improved thermal stability^115^ and SA/hydroxyapatite (HA)/graphene nanoplatelets (GnP) bionanocomposite films for bone tissue engineering.^116^ A hybrid approach combining solvent casting with porogen leaching, using a sacrificial material like salt to create pores, has been used to create porous PPF/GO nanocomposites with a hierarchical architecture suitable for cell infiltration.^117^

#### 3.1.2 Casting with sonication

**Fig. 5:**
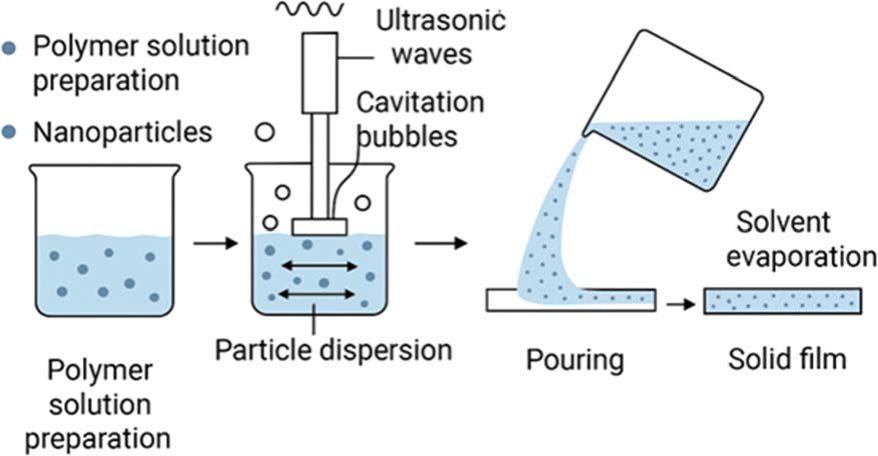
Schematic illustration of the casting process enhanced by ultrasonic sonication.

To overcome the natural tendency of nanoparticles to agglomerate, high-frequency ultrasonic waves are applied to the polymer–graphene solution before casting (Fig. 5). The process, known as sonication, generates acoustic cavitation, which involves the formation and violent collapse of microscopic bubbles. This creates intense localized shear forces that effectively break apart particle aggregates, ensuring a uniform and stable dispersion.^118–120^ This method has been successfully used to fabricate PCL/PGS/GO tubular scaffolds with a uniform porous morphology,^121^ P(3HB)/GnP scaffolds for neuronal studies,^122^ and KGM/GO films with significantly improved mechanical strength (tensile strength of 183.3 MPa) and a well-organized, bioinspired brick-and-mortar structure.^123^

#### 3.1.3 Emulsification

**Fig. 6:**
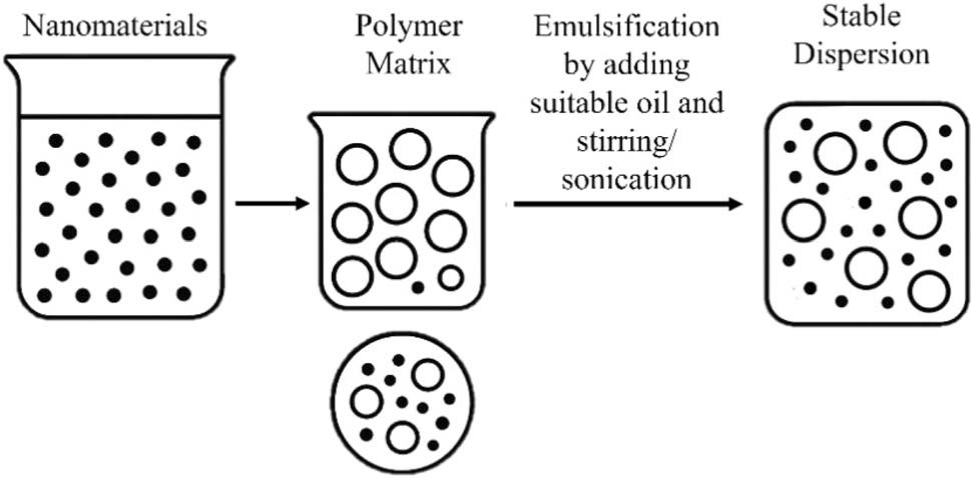
Schematic illustration of emulsification.

This process creates a stable dispersion of two immiscible liquids, such as oil and water, stabilized by a surfactant (Fig. 6). The emulsification step typically involves vigorous stirring or ultrasonication to generate fine droplets of one liquid phase dispersed within the other. Surfactant molecules orient at the interface, reducing interfacial tension and preventing droplet coalescence.^124^ In the context of polymer–graphene systems, the choice of surfactant and solvent pair strongly influences droplet size, stability, and the final morphology of the nanocomposite. Hydrophilic–lipophilic balance governs whether a water-in-oil or oil-in-water emulsion is formed, while the concentration of emulsifier controls droplet size distribution.^125^ Once the emulsion is stabilized, crosslinking or solvent evaporation solidifies the polymer phase, entrapping graphene sheets within spherical domains. The interaction between surfactant functional groups and graphene surfaces can also contribute to dispersion stability, as electrostatic repulsion or steric hindrance suppresses aggregation during processing.^126^ In nanocomposite fabrication, emulsification enables the uniform incorporation of graphene into a polymer matrix, often to form spherical micro- or nanoparticles.^127,128^ For example, a water-in-oil emulsification was used to fabricate starch/agarose/GO nanoparticles for 5-fluorouracil (5FU) delivery, achieving good colloidal stability and drug encapsulation.^129^ A more complex double emulsion (water-in-oil-in-water) method was employed to create a pH-sensitive CS/CMC/GQD/ZnO nanocomposite for quercetin delivery.^130^

### 3.2 Network formation and crosslinking

#### 3.2.1 Crosslinking

**Fig. 7:**
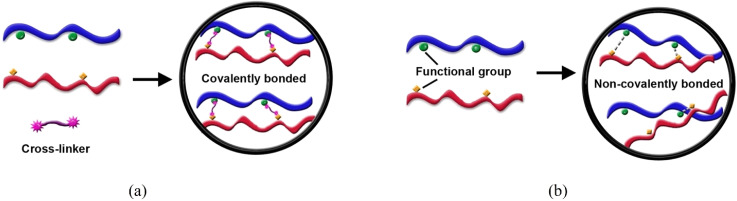
Schematic illustration of polymer crosslinking mechanisms: (a) covalent bonding *via* cross-linkers and (b) non-covalent interactions *via* functional groups.^135^ Ramachandran *et al.*, Cross-linking dots on metal oxides, *NPG Asia Mater.*, 2019, **11**, 19, under the terms of the Creative Commons Attribution 4.0 International Licensee (https://creativecommons.org/licenses/by/4.0/).

This process forms covalent or non-covalent bonds between polymer chains or between polymers and nanofillers, creating a stable three-dimensional network. This network structure is fundamental to hydrogels and enhances the strength, thermal stability, and chemical resistance of the composite (Fig. 7). One common method is chemical crosslinking, which involves the formation of permanent covalent bonds using a chemical agent. For example, hydroxyl-functionalized polymers and polyurethane prepolymers can be used to form robust, crosslinked rGO composite films.^97^ Similarly, a folic acid (FA)–CS–GO quantum dot (GOQD) nanocomposite was synthesized by conjugating FA with CS *via* carbodiimide chemistry using 1-ethyl-3-(3-dimethylaminopropyl) carbodiimide and *N*-hydroxysuccinimide, followed by mixing with GO quantum dots.^131^ In such carbodiimide-mediated reactions, the –COOH groups on GO or FA are activated to form *O*-acylisourea intermediates, which then react with –NH_2_ groups of chitosan, creating stable amide linkages. This covalent grafting not only improves dispersion and interfacial bonding but also alters degradation behavior, since the hydrolytic cleavage of amide bonds is slower compared to ester linkages. In contrast, physical crosslinking relies on weaker, reversible non-covalent interactions like hydrogen bonding, ionic interactions, or hydrophobic associations. GO/γ-poly(glutamic acid) films achieve a strong, nacre-like structure through a combination of hydrogen and ionic bonds (with Ca^2+^ ions).^132^ Similarly, GO–COL scaffolds can be formed *via* pH-dependent electrostatic self-assembly between the negatively charged GO and positively charged COL.^133^ These noncovalent systems are particularly relevant for drug delivery because protonation/deprotonation of functional groups (*e.g.*, –COOH/–COO^−^ or –NH_3_^+^/–NH_2_) under physiological pH changes can reversibly weaken or strengthen the interactions, thereby triggering controlled drug release. Thermal crosslinking is another approach, achieved by heating the material to induce bond formation, often through carbon–carbon networks. In graphene-based biodegradable polymer composites, this method has been shown to significantly improve thermal conductivity and stability.^134^

**Fig. 8:**
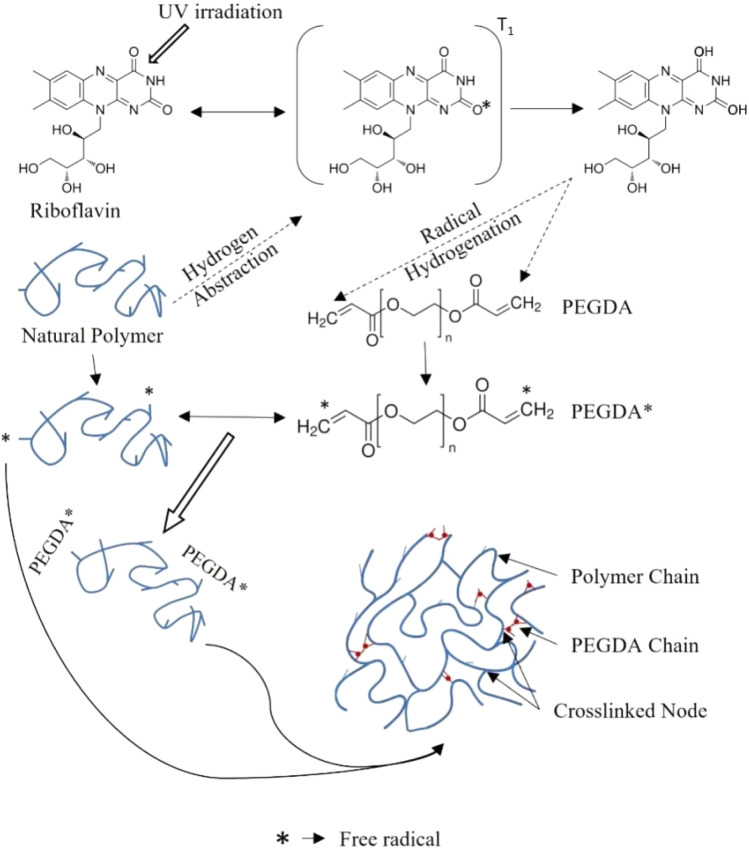
Schematic representation of UV-assisted free radical crosslinking.

Another approach is UV-assisted crosslinking (photocrosslinking), which uses ultraviolet light to activate a photoinitiator, which then generates free radicals that initiate polymerization and crosslinking (Fig. 8). Upon exposure to UV light, photoinitiator molecules absorb photons and are promoted from their ground state to an excited triplet state. In this high-energy state, the photoinitiator can abstract a hydrogen atom from a neighboring polymer chain, generating a polymer radical and a hydrogenated photoinitiator radical. If an auxiliary crosslinking agent is present, the hydrogenated photoinitiator can also induce cleavage of carbon–carbon double bonds within the crosslinker, producing additional radicals. These transient radicals then recombine to form new covalent bonds between polymer chains and/or between polymer and crosslinker segments, resulting in a three-dimensional network.^136,137^ While photoinitiators and crosslinkers are commonly used, some modified polymers, such as methacrylated derivatives (*e.g.*, GelMA, AlgMA), can undergo self-crosslinking under UV light with only a photoinitiator, eliminating the need for an external crosslinking agent.

This technique offers rapid curing and spatial control and has been used to create AESO/PVDF/GO nanocomposites^138^ and to fabricate 3D bioprinted hydrogels from bioinks containing methacrylated polymers (AlgMA, GelMA) and GO for cartilage engineering.^139^ Finally, radiation-induced crosslinking using high-energy sources like γ-rays can generate free radicals on polymer chains and water molecules, initiating polymerization and network formation without the need for chemical initiators, as was done to fabricate sterile GO/(AAc-*co*-SA) interpenetrating network hydrogels.^140^

#### 3.2.2 *In situ* polymerization

**Fig. 9:**
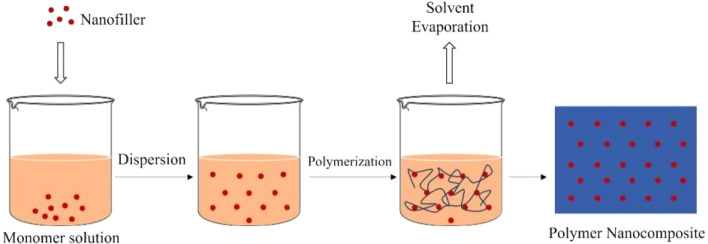
Schematic representation of polymer nanocomposite fabrication *via in situ* polymerization.^144^ Adopted from Basavegowda *et al.*, Advances in functional biopolymer-based nanocomposites for active food packaging applications, *Polymers*, 2021, **13**(23), 4198, under the terms of the Creative Commons Attribution 4.0 International License (https://creativecommons.org/licenses/by/4.0/).

This elegant method involves synthesizing the polymer matrix directly in a solution containing pre-dispersed nanoparticles (Fig. 9). The process begins by uniformly dispersing nanoparticles in a liquid monomer or low-molecular-weight precursor. Polymerization is then initiated through heat, radiation, or chemical initiators, allowing polymer chains to grow around and chemically bond with the nanoparticles. This facilitates the integration of nanoparticles into the polymer network by enabling interactions during chain propagation and network formation.^112^ For instance, when GO or rGO is incorporated, its surface functional groups can form covalent or non-covalent bonds with the polymerizing matrix. In many systems, these groups can act as anchoring sites, leading to interfacial polymerization where the growing polymer chains either initiate from or become physically entangled with the nanoparticle surface.^141^ This results in improved filler–matrix compatibility, reduced agglomeration, and enhanced load transfer efficiency.^142^ Furthermore, in biodegradable polymer systems such as PLA, PCL, or chitosan, *in situ* polymerization can promote uniform dispersion and interfacial adhesion due to hydrogen bonding and polar interactions formed during the growth of the polymer chains. This approach promotes homogeneous filler distribution and allows for the formation of strong interfacial bonds between the growing polymer chains and the nanoparticle surface.^143^

It has been used to create hybrid rGO–multi-walled carbon nanotubes (MWCNT) nanocarriers functionalized with CoNi_2_S_4_ and ZnO, coated with CS and alginate, nanoparticles for co-delivery of drugs and genes,^145^ and to fabricate thiol–maleimide hydrogels incorporating rGO for chemo-photothermal therapy *via* a Michael addition reaction, where hyaluronic acid was functionalized with thiol groups and CS was modified with maleimide groups.^146^ A solvent-free approach using *in situ* polycondensation has also been developed for PGS/gelatin/GO nanocomposites, avoiding the use of potentially toxic solvents.^147^

#### 3.2.3 Free radical polymerization

**Fig. 10:**
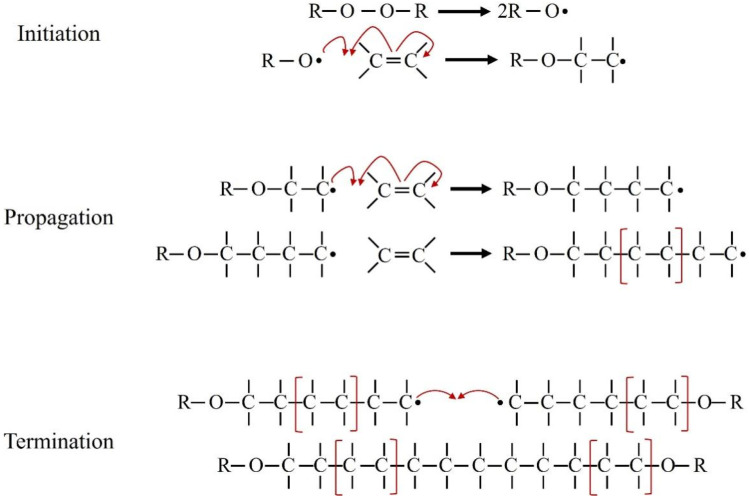
Steps of free radical polymerization.^152^ Adopted from Ribas-Massonis *et al.*, Free-radical photopolymerization for curing products for refinish coatings market, *Polymers*, 2022, **14**(14), 2856, under the terms of the Creative Commons Attribution 4.0 International License (https://creativecommons.org/licenses/by/4.0/).

This common chain-growth process involves initiation, propagation, and termination steps to form polymer chains from monomer precursors (Fig. 10). The process begins with the thermal or photochemical decomposition of initiators such as benzoyl peroxide, AIBN, or ammonium persulfate, generating free radicals that attack the π-bonds of vinyl monomers like acrylamide or methyl methacrylate. This initiates chain growth, where monomers are sequentially added during the propagation phase. Termination occurs through radical recombination or disproportionation, yielding stable polymer chains.^148^ When applied in the synthesis of graphene-based biodegradable nanocomposites, this method enables the polymer matrix to form in direct contact with dispersed graphene derivatives. The presence of oxygen-containing functional groups on materials like GO can influence radical polymerization kinetics, affect the local polymerization environment, and improve filler compatibility by modulating surface energy and dispersion stability.^149^ It is a versatile method for creating hydrogels and is often followed by lyophilization (freeze-drying) to produce highly porous scaffolds. This technique has been used to synthesize multifunctional porous scaffolds by grafting sodium alginate (SA) with acrylic acid (AAc) in the presence of nHA, SiO_2_, and GO,^150^ and to create hybrid scaffolds from GG, AAc, GO, and other nanoparticles for bone tissue engineering.^151^

### 3.3 Advanced and hybrid fabrication techniques

#### 3.3.1 Electrospinning

**Fig. 11:**
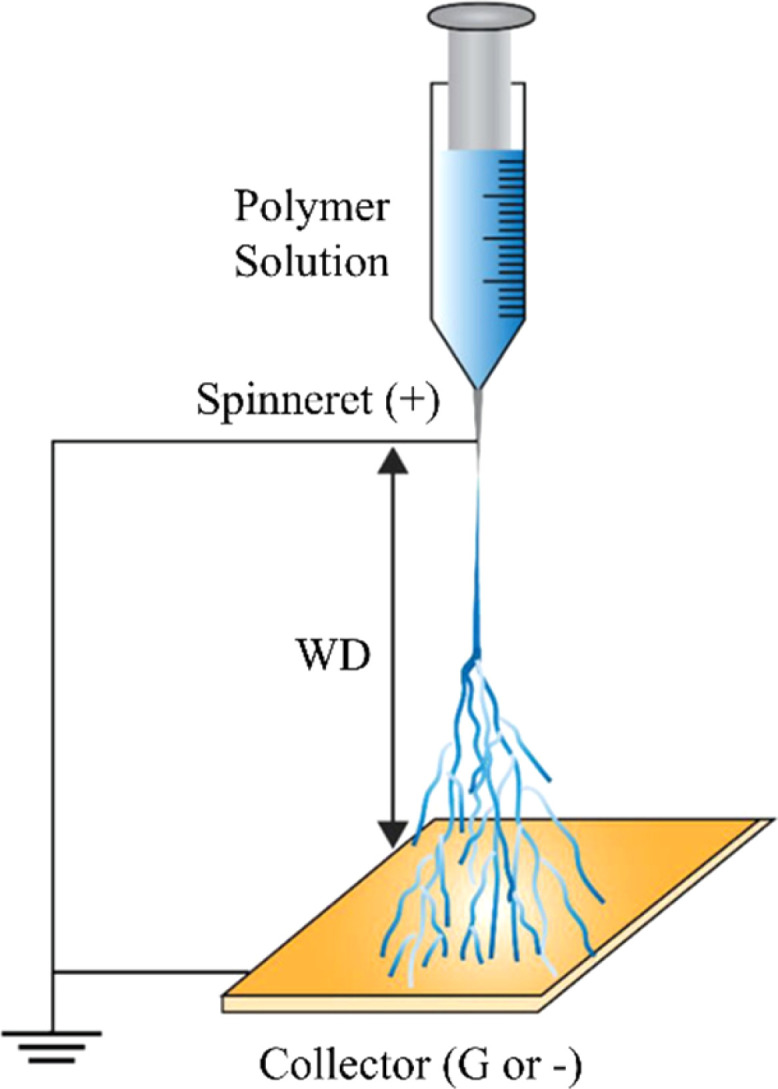
Electrospinning setup for nanofiber fabrication.^161^ Adopted from Antonios Keirouz, Zhe Wang, Vundrala Sumedha Reddy, Zsombor Kristóf Nagy, Panna Vass, Matej Buzgo, Seeram Ramakrishna, and Norbert Radacsi, The History of Electrospinning: Past, Present, and Future Developments, *Adv. Mater. Technol.*, 2023, **8**, 2201723, under the terms of the Creative Commons Attribution 4.0 International License (https://creativecommons.org/licenses/by/4.0/).

This technique uses a high-voltage electric field to draw exceedingly fine nanocomposite fibers (typically nanometer to micrometer scale) from a polymer–graphene solution (Fig. 11). Under high voltage, a polymer–nanofiller solution is ejected as a fine jet from a syringe, stretching and solidifying into nanocomposite fibers with embedded nanofillers as the solvent evaporates. The resulting non-woven nanofibrous mats have a high surface-area-to-volume ratio and a structure that can effectively mimic the native extracellular matrix (ECM).^153,154^ However, the resulting thin mats limit applications to skin or vascular grafts rather than bulk bone regeneration. The success of electrospinning depends on a delicate balance of solution parameters such as viscosity, surface tension, and electrical conductivity,^155^ all of which are strongly influenced by the presence of graphene derivatives. GO, for instance, increases the conductivity of the spinning solution due to its surface charges and polar groups, which can lead to finer fiber diameters and more uniform fiber morphology.^156^ Additionally, uniform dispersion of GO within the polymer matrix is crucial to avoid defects during fiber formation; this often requires prior functionalization or surfactant stabilization.^157^ The interactions between the polymer chains and the nanofillers during electrospinning can influence chain alignment and packing density, ultimately affecting mechanical properties and porosity of the final scaffold. Moreover, residual solvent–filler interactions and rapid solvent evaporation can induce local phase separation or filler aggregation if chemical compatibility is poor.^155^ Electrospinning has been used to fabricate SA/PVA/GnP composite wound dressings,^158^ CS-based nanofibrous mats with GO and carbon quantum dot (CQD)-doped TiO_2_ for accelerated wound healing,^159^ and plasmonic rGO@AuNP–PCL composite scaffolds for synergistic cancer therapy and nerve regeneration.^160^

#### 3.3.2 Wet spinning

**Fig. 12:**
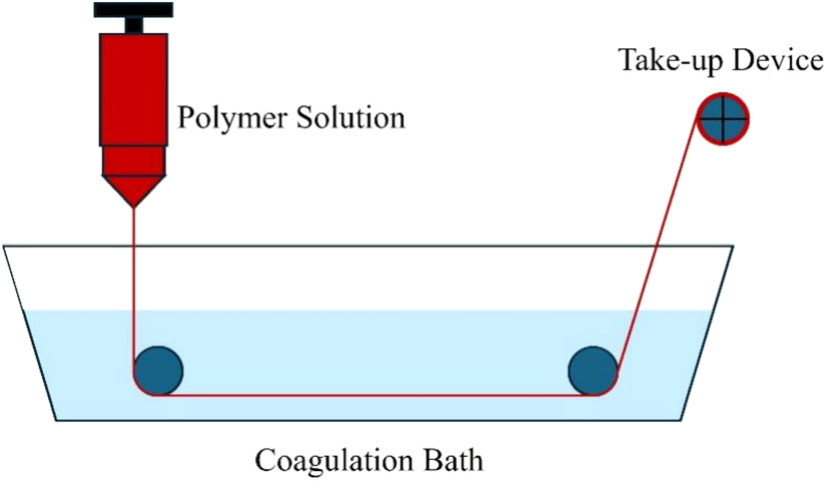
Schematic representation of wet spinning procedure.

In this process, a viscous polymer–graphene solution is extruded through a spinneret directly into a liquid coagulation bath. The solvent exchange between the extruded jet and the bath causes the polymer and any embedded nanofillers to solidify into continuous filaments (Fig. 12).^162^ Wet spinning relies on phase inversion, where the solvent in the polymer solution diffuses into the coagulation bath while nonsolvent molecules simultaneously diffuse into the jet. This induces polymer precipitation, driven by a decrease in solubility and thermodynamic instability of the polymer–solvent system. The presence of graphene-based nanofillers can significantly alter this process by influencing local viscosity, diffusion rates, and nucleation during solidification.^163^ If well-dispersed, graphene derivatives may serve as nucleating agents that guide polymer chain alignment and promote the formation of more crystalline or oriented domains along the fiber axis.^164^ However, poor dispersion or interfacial incompatibility can lead to phase separation or filler aggregation, resulting in heterogeneous fiber morphology.^163^ Surface-functionalized graphene can mitigate these issues by improving interfacial affinity with the polymer matrix, enhancing mechanical integrity.^165^ It has been used to fabricate flexible and electrically conductive alginate–graphene hydrogel biofibers with enhanced mechanical properties and thermal stability.^166^

#### 3.3.3 Freeze-drying (lyophilization)

This dehydration process involves freezing a material (typically a hydrogel) and then reducing the surrounding pressure to allow the frozen water to sublimate directly from a solid to a gas phase.^167^ This gentle removal of solvent preserves the material's delicate porous structure. It is widely used as a crucial post-processing step after hydrogel formation to create stable, highly porous scaffolds suitable for tissue engineering, as demonstrated in the fabrication of CS–GO nanocomposites.^168^ GO–COL composite aerogels further exemplify this approach and were validated through *in vivo* studies.^169^

#### 3.3.4 3D printing and bioprinting

**Fig. 13:**
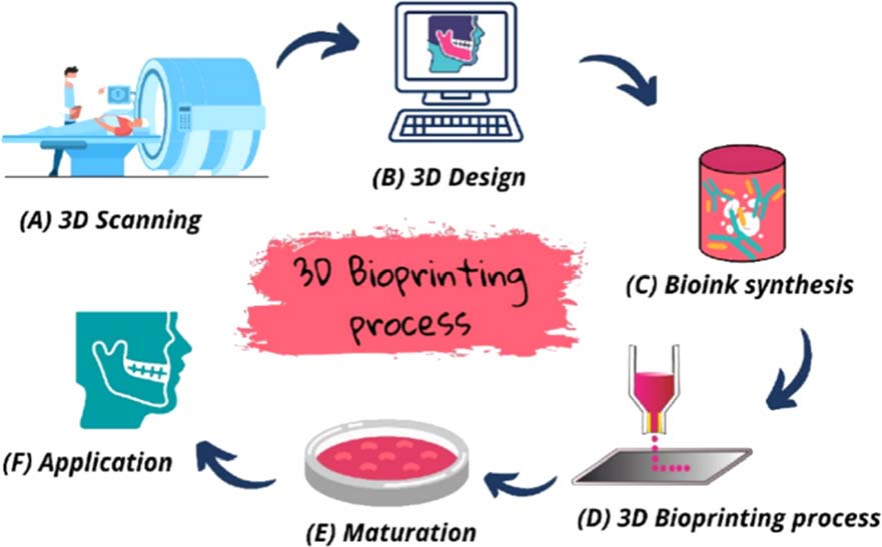
Schematic workflow of the 3D bioprinting process for tissue engineering applications.^176^ Reproduced from Lima *et al.*, 3D bioprinting technology and hydrogels used in the process, *J. Funct. Biomater.*, 2022, **13**(4), 214, under the terms of the Creative Commons Attribution 4.0 International License (https://creativecommons.org/licenses/by/4.0/).

This transformative additive manufacturing technology enables the layer-by-layer fabrication of complex, patient-specific scaffolds with precise control over geometry and porosity. In 3D printing, a thermoplastic polymer–graphene composite is typically melted and extruded as a filament.^170,171^ This has been used to create PCL/GO scaffolds with mussel-inspired coatings^172^ and PLA/GO scaffolds with controlled porosity.^173,174^ 3D printing consistently outperforms traditional methods in shape fidelity and porosity control, though processing temperatures may denature bioactive molecules. 3D bioprinting advances this concept by directly printing “bioinks” containing living cells, biomaterials, and growth factors to construct living tissue structures (Fig. 13).^175^ In bioprinting, patient-specific images (CT/MRI) are converted into 3D models; cells are cultured and mixed with biomaterials to form bioink, which is then printed layer-by-layer and stabilized, followed by maturation to promote tissue formation.^176^ This has been successfully used to create cell-laden hydrogels for cartilage regeneration^139^ and functional, spontaneously beating cardiac rings from rGO-containing bioinks.^177^

#### 3.3.5 Magnetic functionalization

**Fig. 14:**
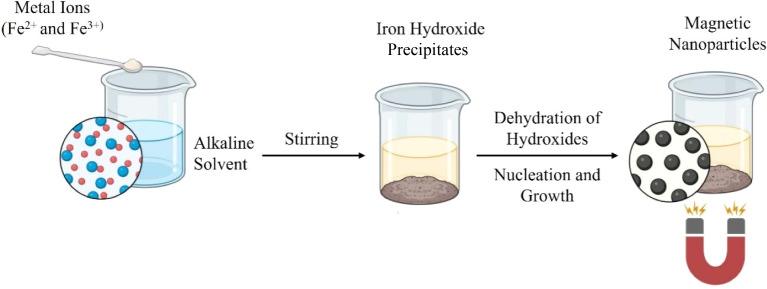
Synthesis of magnetic nanoparticles.^182^ Adopted from Stiufiuc *et al.*, Magnetic nanoparticles: synthesis, characterization, and their use in biomedical field, *Appl. Sci.*, 2024, **14**(4), 1623, under the terms of the Creative Commons Attribution 4.0 International License (https://creativecommons.org/licenses/by/4.0/).

This involves the integration of magnetic nanoparticles, typically iron oxide (Fe_3_O_4_), into the composite to impart magnetic responsiveness. This is commonly achieved *via* an *in situ* co-precipitation method, where ferrous (Fe^2+^) and ferric (Fe^3+^) salts are mixed with GO in an aqueous medium under alkaline conditions. As the pH increases, usually above 10, iron ions react with hydroxide ions to form Fe(OH)_2_ and Fe(OH)_3_, which subsequently undergo nucleation and crystallization into magnetite (Fe_3_O_4_) nanoparticles. The oxygen-containing functional groups on GO act as nucleation and anchoring sites, enabling uniform deposition of nanoparticles onto the graphene surface (Fig. 14). The resulting magnetic BGNs can be guided by external magnetic fields for targeted drug delivery, manipulated for remote-controlled release, or used as contrast agents in magnetic resonance imaging.^178,179^ This method was used to create a multi-responsive SA-*g*-poly(2-hydroxypropyl methacrylamide) (PHPM)/mGO nanocomposite for etoposide delivery.^180^ And a CS-based composite with magnetic GQDs for transdermal microneedle arrays.^181^

## 4 Applications in controlled drug delivery

BGNs are highly effective platforms for controlled drug delivery due to their high drug-loading capacity, enhanced stability, and responsiveness to a range of biological and external stimuli. The large surface area and π-electron system of graphene facilitate high drug loading *via* π–π stacking and hydrogen bonding, while the polymer matrix ensures biocompatibility and biodegradability.^183,184^

### 4.1 Mechanisms of stimuli-responsive release

The “smart” behavior of BGNs stems from their ability to release therapeutic payloads in response to specific triggers. This enables on-demand, site-specific delivery, which is crucial for maximizing therapeutic efficacy and minimizing systemic toxicity.

**Fig. 15:**
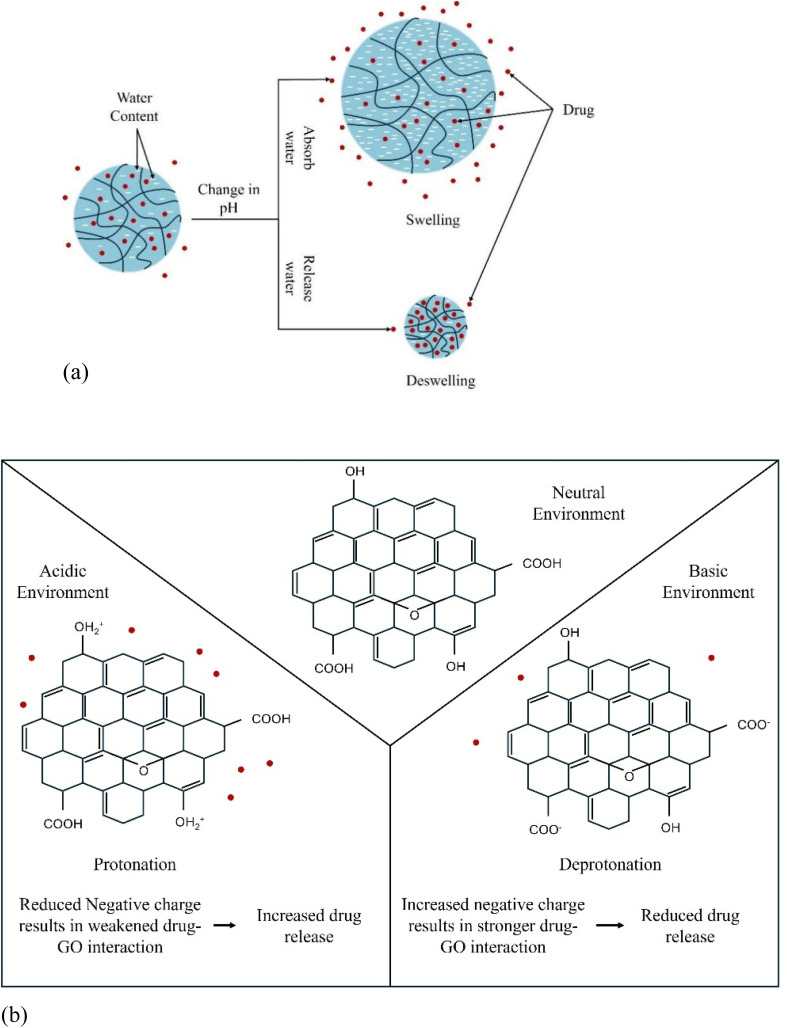
pH-Responsive drug delivery system: (a) swelling and deswelling mechanism, (b) protonation and deprotonation mechanism.

One of the most widely exploited mechanisms is pH-sensitive release, which leverages the pH gradients that exist in the body and in pathological tissues. The mechanism typically relies on the ionization of functional groups within the polymer matrix or on the GO surface. For polymers with acidic (*e.g.*, –COOH) or basic (*e.g.*, –NH_2_) groups, a change in pH alters their charge state, leading to changes in swelling behavior, as illustrated in Fig. 15a.^185^ In an acidic environment, for instance, amine groups on CS become protonated (–NH_3_^+^), leading to electrostatic repulsion between polymer chains. This causes the hydrogel network to swell, increasing the mesh size and facilitating drug diffusion. At the same time, protonation weakens π–π stacking interactions and hydrogen bonding between chitosan chains and GO nanosheets, thereby loosening the carrier network. The protonation/deprotonation of GO's functional groups also plays a key role (Fig. 15b).^186^

At low pH, carboxyl groups are protonated, reducing electrostatic repulsion and weakening the hydrogen bonding interactions that hold the drug, thereby promoting its release. Conversely, at neutral or basic pH, deprotonation enhances interactions (*e.g.*, hydrogen bonding or π–π stacking), stabilizing drug loading and slowing release.^186,187^ Additionally, hydrolysis of ester linkages in polyesters such as PLA and PLGA is accelerated in acidic environments, producing lactic and glycolic acid that further decreases local pH and autocatalyzes matrix degradation.^188,189^ In some systems, pH-sensitive bonds such as Schiff bases and acetals are commonly used in drug delivery systems because they selectively cleave in acidic environments. Under low pH, Schiff bases hydrolyze due to protonation of the imine nitrogen, allowing water to attack and cleave the bond (*e.g.*, R–CH=N–R′ + H_2_O → R–CHO + R′–NH_2_), leading to controlled drug release in acidic environments.^190^ For instance, imine (C=N) bonds in Schiff base linkages undergo protonation followed by nucleophilic attack by water, whereas acetals (C–O–C) are cleaved through acid-catalyzed oxonium ion intermediates, both providing predictable release profiles in tumor-like acidic conditions.^191,192^

**Fig. 16:**
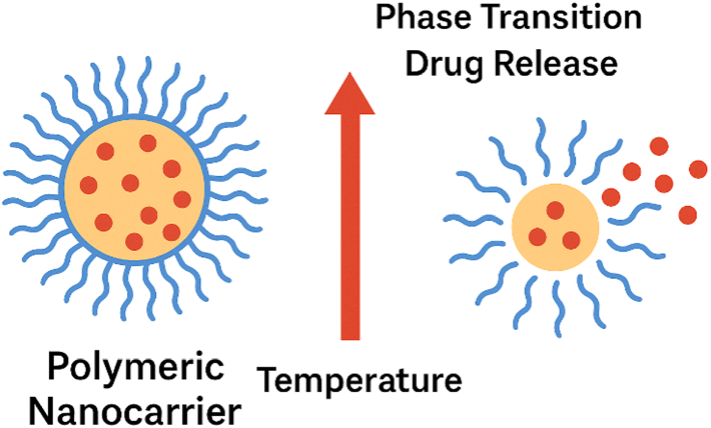
Schematic illustration of thermo-responsive drug release: at temperatures below the LCST, the nanocarrier remains swollen and retains the drug; upon heating above the LCST, the polymer network collapses, triggering.

Another important mechanism is thermo-responsive release, which uses temperature as a trigger (Fig. 16). It is often designed using polymers like poly(*N*-isopropylacrylamide) (PNIPAAm) that exhibit a lower critical solution temperature (LCST). Below the LCST, the polymer is hydrophilic and swollen, retaining the drug. Above the LCST, the polymer undergoes a phase transition, this transition arises from disruption of hydrogen bonding between PNIPAAm's amide groups and water, leading to hydrophobic association within the network, which squeezes out the encapsulated drug.^193,194^ Graphene's excellent photothermal properties can be harnessed here; when incorporated into a thermosensitive polymer matrix, near-infrared (NIR) irradiation can be used to remotely heat the BGN above its LCST, triggering on-demand drug release.^195^

Enzyme-responsive release offers high specificity by exploiting enzymes that are overexpressed in pathological tissues, such as matrix metalloproteinases (MMPs) in the tumor microenvironment. In the context of BGN, this responsiveness can be engineered by incorporating enzyme-cleavable peptide linkers either between the therapeutic agent and the nanocarrier or within the polymer backbone itself. Upon exposure to the target enzyme, specific peptide bonds (–CO–NH–) are hydrolyzed (R–CO–NH–R′ → R–COOH + H_2_N–R′), resulting in cleavage of the linker or degradation of the polymer matrix. This cleavage destabilizes the nanocomposite structure, triggering the controlled release of the encapsulated or conjugated drug. In particular, hydrophilic oxygen-containing groups on GO and surface functionalities of the polymer can enhance enzyme accessibility and interaction, facilitating more efficient degradation and payload release payload.^196–198^

**Fig. 17:**
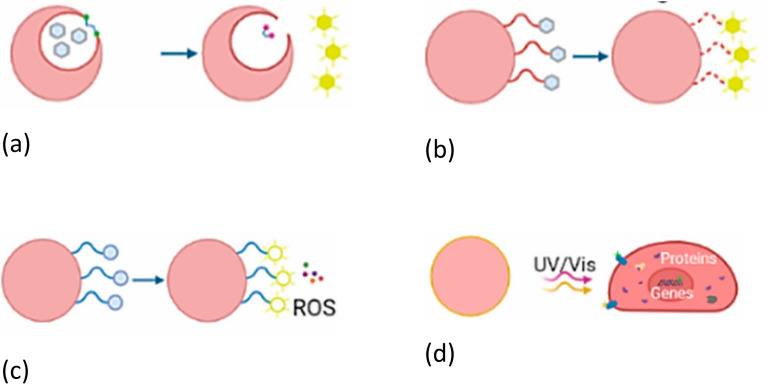
Schematic representation of different photosensitive drug release mechanisms: (a) photoisomerization, (b) photocleavage, (c) photosensitization, and (d) photoactivation.^202^ Adopted from Fernández *et al.*, Advances in functionalized photosensitive polymeric nanocarriers, *Polymers*, 2021, **13**(15), 2464, under the terms of the Creative Commons Attribution 4.0 International License (https://creativecommons.org/licenses/by/4.0/).

Photo-responsive release, illustrated in Fig. 17, can be achieved through several pathways beyond the photothermal effect. Photo-cleavable linkers can be incorporated into the BGN structure; upon irradiation with a specific wavelength of light, these bonds break, releasing the drug (Fig. 17b).^199^ Alternatively, photoisomerization (Fig. 17a) uses molecules like azobenzene that change conformation under light, altering the carrier's structure and porosity to allow drug diffusion.^200^ Additionally, photosensitization involves a photosensitizer that, upon light exposure, produces reactive oxygen species (ROS) causing cell damage or drug release, forming the basis of photodynamic therapy (Fig. 17c).^201^ Similarly, photoactivation uses light to convert inactive prodrugs into active therapeutic forms at the target site (Fig. 17d).

**Fig. 18:**
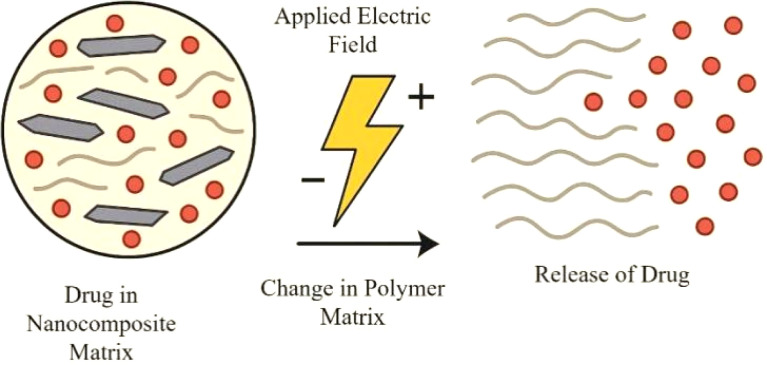
Electro-responsive release mechanism.

Finally, electro- and magnetically-triggered release offer remote control. Graphene's conductivity allows for electro-responsive systems where an applied electric field can induce conformational changes or electrophoretic movement to trigger release (Fig. 18).^203^ For magnetic release, incorporating magnetic nanoparticles allows an external oscillating magnetic field to induce localized hyperthermia or mechanical deformation of the scaffold, both of which can drive drug release.^204,205^

A comparative summary of these release mechanisms is provided in Table 3.

**Table 3:** Comparison of drug release mechanisms in biodegradable polymer–graphene nanocomposites

Release mechanism	Advantages	Disadvantages	Usage frequency in BGN
pH-Sensitive	• Targeted release in acidic microenvironment	• Off-target release in any acidic site (*e.g.* stomach)	Common
	• Minimal release at normal pH	• Requires precise tuning of polymer p*K*_a_	
	• Polymer swelling at low pH enhances release	• Possible drug degradation in strong acid	
Thermo-responsive	• On-demand release *via* external heating	• Risk of tissue damage if overheated	Moderately used
	• Synergistic with hyperthermia-based therapy	• Requires effective heat delivery to target site (depth/penetration issues)	
Enzyme-responsive	• High specificity if target enzyme is unique	• Complex design (requires enzyme-cleavable linkers)	Less common
	• Activated under mild physiological conditions	• Variability in enzyme expression levels (patient-to-patient)	
Photo-responsive	• Precise spatiotemporal control	• Phototoxicity/heating of healthy tissue is possible	Less common
	• Deep NIR penetration enables efficient photothermal ablation	• Limited penetration for visible/UV light	
Electroresponsive	• Fine control over drug release by modulating electrical signal	• Requires conductive materials	Less common
	• Can be easily integrated into implantable devices	• Risk of electrochemical reactions and tissue irritation	
		• Limited penetration depth in tissue	
Magnetically triggered	• Remote, non-invasive control	• Requires incorporation of magnetic nanoparticles	Moderately used
	• Enables localized hyperthermia and mechanical actuation	• Risk of local overheating or unintended tissue exposure	
	• Magnetic targeting enhances accumulation at desired site		

### 4.2 Drug diffusion mechanisms

**Fig. 19:**
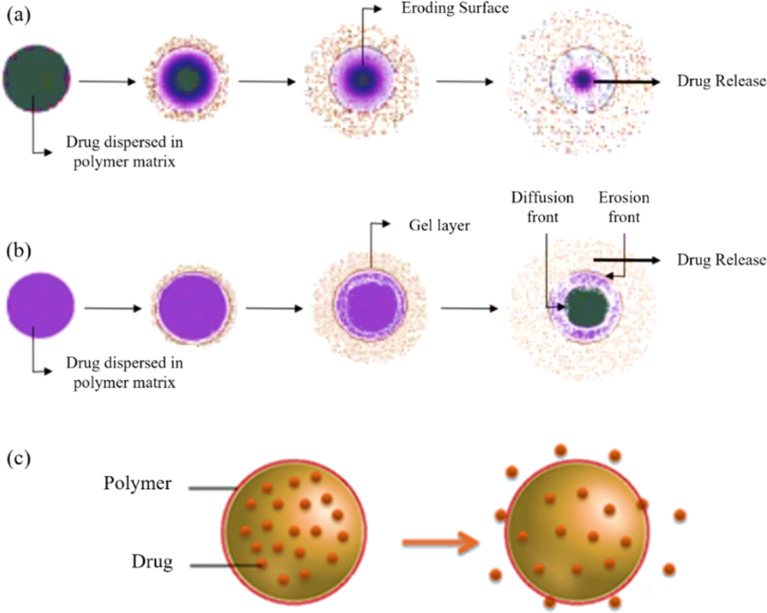
Illustration of drug release from diffusion-controlled delivery systems, where the drug is uniformly distributed in: (a) an erodible polymer matrix, (b) a hydrophilic, swellable polymer matrix, and (c) a reservoir-type system.^211,213^ Adapted from Joseph *et al.*, Emerging Bio-Based Polymers from Lab to Market: Current Strategies, Market Dynamics and Research Trends, *C*, 2023, **9**(1), 30, and Hossain *et al.*, Scope of bio-based nanoparticle targeted through the cancer zone to deactivate cancer affected cells, *Chem. Phys. Impact*, 2023, **6**, 100180, both under the terms of the Creative Commons Attribution 4.0 International License (https://creativecommons.org/licenses/by/4.0/).

In addition to stimuli-responsive release, the passive diffusion of drugs from the BGN matrix is a fundamental process. The rate of release is governed by the concentration gradient and the physical properties of the matrix.^206^ In erodible matrices, the drug is released as the polymer matrix gradually degrades or dissolves from the surface inward, exposing new layers of drug over time (Fig. 19a). Enzymatically degradable polymers (*e.g.*, collagen, gelatin) coupled with GO generally degrade faster than synthetic polyesters, highlighting their suitability for soft tissue repair rather than long-term implants.^207^ In contrast, hydrophilic matrices swell upon contact with aqueous fluids, forming a gel layer (Fig. 19b). The drug must then diffuse through this viscous gel layer, and the release rate is controlled by the rate of swelling and the thickness of the gel.^208^ Polyester-based BGNs degrade primarily through hydrolysis, which is slowed by graphene reinforcement due to reduced water uptake and chain mobility.^209^ Interestingly, electrostatic polymer–graphene interactions accelerate degradation in acidic microenvironments by destabilizing the network, a feature exploited in tumor-targeted release systems.^210^ In reservoir-type systems, the drug is contained within a core that is encapsulated by a rate-controlling polymer membrane. The drug diffuses through this membrane to the external environment (Fig. 19c).^211^ Comparisons show that covalently bonded systems maintain mechanical stability during degradation, while non-covalent systems show early-stage swelling and burst release.^212^

The release kinetics in these systems are often described by mathematical models, such as Fickian diffusion, where release is proportional to the square root of time, or more complex non-Fickian (anomalous) models when polymer relaxation and drug diffusion rates are comparable.^214^ This evidence suggests that degradation can be fine-tuned by adjusting graphene loading and interfacial chemistry rather than polymer choice alone.

### 4.3 Applications in cancer therapy

BGNs have shown significant promise in cancer therapy by enabling targeted delivery and controlled release, thereby enhancing efficacy and minimizing side effects. In the context of breast cancer, multiple studies have targeted MCF-7 and MDA-MB-231 cells. A GO–CS–FA nanobiocomposite co-loaded with camptothecin and diindolylmethane achieved 95.7% inhibition of MCF-7 cells and showed enhanced *in vivo* bioavailability.^215^ A starch/agarose/GO system for 5FU delivery demonstrated accelerated release at pH 5.4.^129^ Combining chemo- and photothermal therapy, a dopamine–rGO hydrogel loaded with doxorubicin (DOX) reduced MCF-7 viability to 21% under NIR irradiation.^146^ For lung cancer, GO-based systems have been developed to target A549 and H1299 cells. An folic acid–CS–GOQD nanocomposite showed pH-responsive DOX release and folate receptor-mediated uptake, inducing apoptosis selectively in cancer cells.^131^ A multi-responsive SA-*g*-PHPM/mGO system for etoposide delivery was triggered by pH, NIR light, and magnetic fields, enabling precise, multi-modal therapy that led to a sharp reduction in H1299 cell viability.^180^ BGNs have also been applied to other cancers. For colon cancer, a CS/GO/TiO_2_/escin nanocomposite demonstrated significant cytotoxicity against COLO 205 cells through TiO_2_-induced reactive oxygen species (ROS) generation.^216^ For cervical cancer, an FA-functionalized gelatin-coated GO nanocarrier delivered chlorambucil with pH-responsive release kinetics.^217^ For melanoma, an rGO–5FU–CMARX hydrogel showed pH-dependent 5FU release and strong antibacterial activity.^218^

### 4.4 Treatment of infectious and other diseases

Beyond cancer, BGNs are being developed for a range of therapeutic areas. For infectious diseases, nanoengineered self-assembling peptide-based hydrogels incorporating GO have been used for the sustained release of drugs against tuberculosis (isoniazid), fungal infections (amphotericin B), and bacterial infections (ciprofloxacin).^219^ In the treatment of neurological disorders, double-network hydrogels composed of amino-acid-based networks reinforced with polyacrylamide (PAAm), PNIPAAm, agarose, or low-gelling agarose, containing GO or carbon nanotubes (CNTs) as photothermal agents, have been used for the on-demand, NIR light-triggered release of baclofen to treat severe spasticity.^220^ For gastrointestinal conditions, a CS–GQD/sodium salicylate@CMC hydrogel bead system was designed for oral delivery, protecting the drug from the acidic stomach and enabling controlled release in the intestines.^221^ Finally, in ocular drug delivery, a CS–GQD nanocomposite demonstrated enzyme-triggered release of latanoprost for glaucoma treatment, responding to lysozyme in tear fluid.^222^

A summary of selected studies on BGNs for controlled drug delivery is presented in Table 4.

**Table 4:** Applications of BGN systems in controlled drug delivery

Polymer matrix	Graphene type and additives	Application (target, drug, mechanism)	Observed outcomes (*e.g.* release%, viability, responsiveness)	References
CS	rGO–5FU blended with CMARX (from *Plantago ovata*)	Melanoma therapy and wound healing; pH-responsive 5FU delivery	93.1% release at pH 7.4; sustained release at pH 6.4; promotes skin cell proliferation; antibacterial *vs. S. aureus*, *P. aeruginosa*	218
FA-functionalized CS	GOQDs; DOX	Cancer therapy (A549, SH-SY5Y), folate-receptor targeting	57% DOX release at pH 5.5 *vs.* 12% at pH 7.4; nuclear fragmentation; less than 5% hemolysis; selective cytotoxicity	131
CS/CMC	GQD beads in CMC, sodium salicylate	Oral delivery, inflammation therapy (pH-responsive)	Minimal release at pH 1.2; enhanced at pH 6.8 and 7.4; more than 70% HT29 cell viability	221
Alg/AAc	GO	Colon-specific drug delivery; pH-responsive cefadroxil release	Minimal release at pH 1 (stomach); sustained release at pH 7 (intestine); GO reduces burst release and regulates swelling	140
SA/K-CG	rGO	Amoxicillin delivery for wound treatment	94% loading efficiency; 26% release at pH 5.5 *vs.* 34% at pH 7.4 over 95 h; Fickian release; strong antibacterial activity	223
SF	GO (0.5–3 wt%)	Controlled drug delivery (rhodamine B) and tissue engineering scaffolds	β-Sheet content peaked at 1.0% GO then declined; reduced burst release and sustained RhB release; faster degradation with higher β-sheet	114
2-HEC	HCl/HNO_3_-modified graphene	Nanocomposite films for potential biomedical applications (drug delivery, biosensors)	Enhanced thermal stability (11 °C increase in *T*_max_); hydrophilic transformation of graphene	115
CS	GO–CS–FA decorated with camptothecin and diindolylmethane	Breast cancer (MCF-7); targeted co-delivery system	95.67% inhibition (MCF-7); enhanced bioavailability (AUC ∼ 33 858 ng mL^−1^ h^−1^); low renal/liver toxicity	215
Gelatin-coated GO	FA-functionalized rGO	Cervical cancer (Siha), chlorambucil delivery	82% release at pH 1.2, 62.1% at pH 5.4, 43.7% at pH 7.4; IC50: 125.9 µg mL^−1^ (*vs.* 86 µg mL^−1^ for free drug)	217
Starch/agarose	GO	Breast cancer (MCF-7), 5FU delivery	High 5FU encapsulation (∼87.3%); significant MCF-7 growth inhibition; acidic pH triggers release (targeted tumor delivery)	129
SA grafted with PHPM	mGO, Fe_3_O_4_-decorated GO	Lung cancer (H1299), etoposide delivery	Triple stimuli-responsive release (pH 5.5, NIR, magnetic); cell viability drops to ∼19.1% (H1299); acid/NIR/magnet enhance release	180
CS/CMC	GQDs; ZnO nanoparticles	Brain cancer (U-87 MG), quercetin delivery	49% inhibition of U-87 MG (cancer cells); 85% viability in L929 (normal); pH-sensitive release (*t*_1/2_ ≈ 72 h)	130
CS (folate-functionalized)	rGO; NiO nanoparticles; FA targeting ligand	Lung (A549) and breast (MCF-7), DOX delivery	DOX release ∼98.6% at pH 5 *vs.* 9.6% at pH 7.4; A549 viability 12.3%, MCF-7 7.1%; low zebrafish toxicity	224
CS	rGO, Pd nanoparticles	Colon cancer (HT-29), dual-drug system (5FU + another)	∼95–98% encapsulation; pH-sensitive release; IC50: 9.87 µg mL; Fickian diffusion kinetics	225
CS (folic acid-modified)	GO nanoscrolls; FA-functionalization; DOX + caffeic acid	Lung carcinoma (A549), co-delivery of DOX and caffeic acid (CA)	DOX release ∼83% at pH 5 *vs.* 71% at pH 7.4; selective apoptosis in A549, high viability in HEK293 (normal)	226
CS	GO; TiO_2_ nanoparticles; escin	Colon cancer (COLO 205)	IC_50_ ≈ 22.7 µg mL^−1^ (COLO 205); induces ROS-mediated apoptosis; minimal toxicity to normal cells	216
CS	GQDs	Ocular drug delivery; latanoprost for glaucoma; real-time tracking *via* photoluminescence	“On–Off–On” photoluminescence response with lysozyme; more than 80% cell viability; protective effect against H_2_O_2_-induced oxidative damage in human corneal epithelial cells	222
CS/alginate	rGO; MWCNTs; CoNi_2_S_4_ and ZnO nanoparticles	Co-delivery (HeLa/HEK-293), DOX and pCRISPR	pH-Responsive sustained DOX release (more in acidic environment); enhanced cellular uptake; improved therapeutic efficacy (co-delivery)	145
Thiol–maleimide hydrogel	Dopamine–rGO; DOX	Breast cancer (MCF-7), chemo-photothermal therapy	MCF-7 viability ∼21% under NIR; pH-sensitive DOX release accelerated by NIR (Δ*T* ≈ 22 °C); targeted chemo/photothermal effect	146
Self-assembling peptide hydrogel	GO; (with antibiotics: isoniazid, amphotericin B, ciprofloxacin)	Sustained delivery of TB/antifungal/antibacterial drugs	Sustained, controlled release of each loaded drug (isoniazid, amphotericin B, ciprofloxacin); good biocompatibility	219
Double-network hydrogel (PAAm/agarose or PNIPAAm/agarose)	GO, CNT (photothermal agents); Fmoc-protected amino acids	Neurological (spasticity), baclofen release (NIR-triggered)	NIR-triggered baclofen release; inclusion of GO/CNT enables photothermal actuation (CNT-containing gel shows highest release efficiency)	220
CS	MrGO (Fe_3_O_4_@RGO) + Pluronic F127; α-mangosteen	Breast cancer (MCF-7), α-mangosteen delivery	Faster release at pH 5.5 (tumor-like) *vs.* 7.4; magnetic field enables targeting; inhibited MCF-7 proliferation	227
CS	mGQDs	Transdermal microneedle arrays; electrically triggered drug delivery	96.4% drug release under electrical stimulation *vs.* 25.7% passive diffusion; detachable design with PEG base for rapid fluid response	181
PCL	GO, Fe_3_O_4_, TRAIL, DOX	Magnetically-triggered cancer therapy (scaffold system)	Magnetic stimulation enhances drug release; dual action therapy; localized delivery; *in vivo* validation not provided	228
Aminated CMC	GO; Fe_3_O_4_ magnetic nanoparticles; curcumin	Breast cancer (MDA-MB-231), curcumin delivery	Enhanced cytotoxicity *vs.* free curcumin; rapid curcumin release at pH 5.5 *vs.* slower at 7.4; magnetic targeting improves localization	186
CMC	N-doped graphene, imatinib mesylate	pH-Responsive cancer therapy (simulated tumor microenvironment)	∼74% loading at pH 7.0 in 3 h; ∼58% release at pH 4.0; reduced release at neutral/basic pH	229

## 5 Applications in tissue engineering

In tissue engineering, scaffolds provide a three-dimensional framework that mimics the native ECM, offering physical support and biological cues to guide cell adhesion, proliferation, and differentiation for the regeneration of new tissue.^230,231^ BGNs are excellent scaffold materials because the biopolymer component provides essential biocompatibility and biodegradability, while the graphene component significantly enhances mechanical strength, stability, and bioactivity.^232–235^

### 5.1 Scaffold design principles and tissue engineering strategies

The success of a tissue engineering strategy depends on the careful design of scaffolds that can orchestrate a complex series of biological events. This can be achieved through two primary approaches: *in vitro* and *in vivo* tissue engineering.

**Fig. 20:**
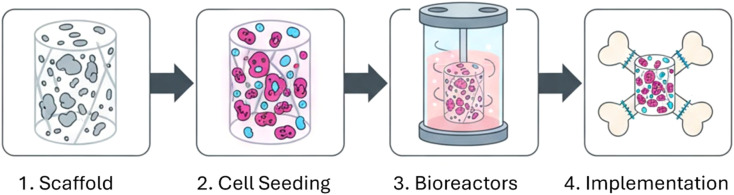
Steps of *in vitro* tissue engineering.

The classic approach is *in vitro* tissue engineering, which involves creating tissues outside the body in a controlled laboratory setting (Fig. 20). The process begins with isolating cells from a patient or donor and seeding them onto a pre-fabricated 3D scaffold. This cell-seeded construct is then cultured in a bioreactor, which provides a dynamic environment with nutrients, oxygen, and mechanical or electrical stimuli to promote cell proliferation, differentiation, and ECM production. Once the engineered tissue reaches a desired level of maturity, it is implanted into the patient.^236–238^ However, implantation into the human body is not always the ultimate or mandatory step, as *in vitro* tissue constructs are also crucial for evaluating scaffold performance, studying cell–material interactions, modeling diseases, and testing drugs in a physiologically relevant environment.

**Fig. 21:**
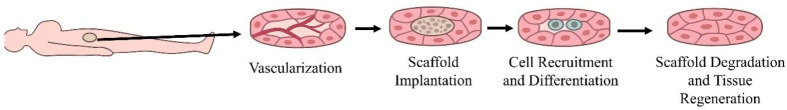
Steps of *in vivo* tissue engineering.

In contrast, *in vivo* tissue engineering leverages the body's own regenerative capacity by using it as a natural bioreactor (Fig. 21). A biomaterial scaffold, often loaded with growth factors or other signaling molecules, is implanted directly into the site of injury. The scaffold is designed to recruit endogenous stem or progenitor cells from surrounding tissues. These recruited cells then populate the scaffold, differentiate into the appropriate cell types, and regenerate the damaged tissue *in situ*. As the new tissue forms, the scaffold gradually degrades and is replaced.^239,240^ However, studies in this area remain comparatively limited, as this approach requires significant prior research and validation to ensure safety and efficacy.

Most of the studies discussed in the following sections are thereby focused only up to various stages of the *in vitro* process. Some involve scaffold fabrication alone, others extend to cell seeding and bioreactor-based culture, while only a few report implantation into a living body. Understandably, *in vivo* studies are far less common than *in vitro* ones due to the extensive safety and regulatory requirements involved. Regardless of the approach, the scaffold's properties are paramount. It must be biocompatible, biodegradable at an appropriate rate, and possess sufficient mechanical integrity. Furthermore, its architecture must feature an interconnected porous network to allow for cell infiltration and nutrient transport.^230,231,241^ The incorporation of graphene into biopolymer scaffolds can enhance all these properties, improving mechanical strength while providing a surface that promotes cell adhesion and guides differentiation.^234,235^

### 5.2 Bone and cartilage regeneration

BGNs are particularly well-suited for bone and cartilage tissue engineering due to their ability to provide robust mechanical support and promote chondro/osteogenesis. In terms of osteogenic potential, numerous studies have shown that GO-based composites enhance osteoblastic differentiation. For example, PGA-*co*-PF/GO/HA electrospun scaffolds significantly increased alkaline phosphatase activity in MG63 cells,^78^ and SA/HA/GnP films promoted apatite-like mineral formation in simulated body fluid.^116^ For load-bearing applications, the reinforcing effect of graphene is critical. PCL scaffolds reinforced with 3% graphene achieved a compressive modulus of 136.74 MPa,^242^ while PLLA scaffolds incorporating 12% GO–hydroxyapatite reached a compressive strength of 21.52 MPa.^243^ In the challenging area of cartilage regeneration, biomimetic hydrogels composed of alginate, gelatin, chondroitin sulfate, and GO have been 3D bioprinted with mesenchymal stem cells, inducing intrinsic chondrogenic differentiation without exogenous growth factors.^139^

### 5.3 Neural and cardiac tissue engineering

For neural tissue, scaffolds must support cell viability and provide electrical cues to guide regeneration. Graphene's conductivity is a major advantage. GO–COL and rGO–COL scaffolds supported extensive Schwann cell spreading and attachment,^133^ while P(3HB) scaffolds containing GnP restored physiological firing patterns in cultured neurons.^122^ For cardiac tissue, which relies on electrical signal propagation for synchronized contractions, a 3D bioprinted cardiac “BioRing” fabricated from a GelMA/AlgMA/rGO bioink successfully mimicked key aspects of native heart tissue, including spontaneous and synchronous beating.^177^

### 5.4 Skin and wound healing

BGNs can accelerate wound healing by acting as both protective wound dressings and pro-regenerative templates. Electrospun mats of SA/PVA/GnP loaded with curcumin provided sustained antimicrobial and antioxidant effects,^158^ while GO–CS/PVP membranes enhanced skin wound repair in rat models, showing 33% faster wound closure than sterile gauze.^244^ For skin regeneration, CS–GO scaffolds implanted subdermally in rats supported tissue encapsulation and vascularization.^168^ Furthermore, PLLA nanofiber scaffolds coated with polydopamine and carbon nanomaterials such as GO and CNTs have been shown to exhibit piezoelectric behavior, suggesting they could generate therapeutic electrical cues to accelerate healing in response to body movement.^245^

A detailed summary of selected studies on BGNs for tissue engineering is presented in Table 5.

**Table 5:** Applications of BGN systems in tissue engineering

Polymer matrix	Graphene type and additives	Applications (target tissue and role)	Observed outcomes	References
PGA-*co*-PF	GO, HA	Bone – electrospun scaffold	Increased alkaline phosphatase activity; osteoblastic differentiation; more than 5 times protein adsorption; greater than 98% metabolic activity	78
COL	GO	Neural – scaffold for nerve tissue engineering	Schwann cell adhesion/spreading; 3D porous (20–100 µm); mechanically stable; cell infiltration	133
AESO/PVDF blend (UV-crosslinked)	GO; curcumin	Bone – bone tissue scaffold	Semi-crystalline matrix with enhanced stiffness; antibacterial against common pathogens; supports osteoblast viability	138
Alginate/gelatin/chondroitin sulfate bioink	GO	Cartilage – 3D bioprinted scaffold	Promoted intrinsic chondrogenic differentiation of stem cells; high cell viability; improved ECM synthesis	139
GelMA/AlgMA bioink	rGO	Cardiac – 3D bioprinted cardiac tissue scaffold	Supported cardiomyocyte viability, alignment, and beating; enhanced electrical conductivity for synchronized activity	177
Type I COL	GO (0.05–0.2% w/v)	Bone – aerogel scaffold for cranial defects	Enhanced compressive modulus (0.20–0.51 MPa); superior biomineralization (Ca/P = 1.67); 1.5× increased bone volume *in vivo*; improved rat bone marrow mesenchymal stem cell proliferation	169
SA	GnP; HA	Bone – scaffold for bone regeneration	Nearly doubled tensile strength at 0.5% GP; promoted apatite-like mineralization; excellent biocompatibility and biodegradability	116
CS (anisotropic membrane)	GO; HA	Bone – bone-mimicking membrane scaffold	Improved early osteoblast viability and long-term growth; enhanced cell spreading and adhesion; favorable microenvironment *via* GO/HA	246
PPF	GO nanoribbons/nanoplatelets	Bone – porous scaffold for bone regeneration	26% modulus increase; cell infiltration; ECM formation; enhanced osteoblast viability	117
PCL	rGO–Ag nanoparticles	Bone – film scaffold	Increased mechanical/electrical properties; promotes stem cell differentiation; antimicrobial	247
CS beads	GO, TiO_2_ nanoparticles	Bone – injectable beads	Less than 1% resorption over 90 days; osteoconductivity; COL type I formation; enhanced crystallinity	248
PCL/PGS tubular scaffold	GO	Nervous/vascular/renal – nerve conduit	About 84% fibroblast viability; enhanced compressive modulus and thermal stability; channel-like porosity aiding regeneration	121
P(3HB)	GnP	Neural – conductive neuronal scaffold	Restored physiological neuronal firing patterns; increased responsiveness at low stimulus; dense neuronal network formation	122
CS (GLA-crosslinked beads)	GO; TiO_2_ nanoparticles; blackberry extract	Bone – injectable bead scaffold	Strong *in vivo* biocompatibility; stimulated new bone formation and mineral deposition; COL fiber development	249
KGM	GO	Scaffold through solvent casting	Young's modulus: 16.8 GPa; tensile strength: 183.3 MPa; over 90% cell viability; strong cell adherence; bioactive and biocompatible	123
PLGA	GO, MoS_2_ nanoplatelets	Bone – porous scaffold	20–27% early bone regeneration; minimal inflammation; enhanced mechanical strength	250
CS	GO	Skin – scaffold	Around 78 µm porosity; improved vascularization and healing; mild inflammation	168
SA/PVA	GnP; curcumin	Skin – electrospun wound dressing	Controlled curcumin release (∼80% in 24 h); combined antimicrobial and antioxidant effects; supports tissue regeneration	158
PVA + CS nanofibers	GO; CQD-doped TiO_2_	Skin – nanofiber wound healing scaffold	Accelerated wound closure (greater than 93% in 14 days); promoted fibroblast migration; antibacterial against gram-positive and negative bacteria	159
CS/PVP nanofibers	GO	Wound healing – nanofibrous mat	*In vitro* study: more cell viability (40%); faster wound closure (33%); mimics ECM; water-permeable	244
Zein nanofibers	GO, curcumin	Wound healing – dressing	*In vitro* study: biphasic drug release; fibroblast proliferation; low swelling, controlled CUR release	251
PLGA/gelatin	GO	Bone – electrospun scaffold	Increased ALP, RUNX2, calcium deposition; ECM-like morphology; supports osteogenic differentiation	252
PCL	rGO, AuNPs	Neural – aligned nanofiber scaffold	Improved neurite outgrowth (2.5×); 90% Schwann cell viability; NIR-induced tumor cell ablation	160
Gelatin	GO	Bone – electrospun scaffold	Improved Young's modulus (70%) and tensile strength (200%); supports osteoblast proliferation	253
Alginate	GO	Muscle – biofiber scaffold	*In vitro* study: increased tensile strength/modulus; C2C12 myoblast viability and differentiation; myogenic morphology	166
PLLA	GO–HA hybrid composite	Bone – load-bearing scaffold	Increased compressive strength (∼21.5 MPa) and modulus (∼5 GPa); apatite layer formation in simulated body fluid; excellent osteoblast compatibility	243
CS–PPPOEMA	GO–Ag	Wound healing – scaffold	Improved wound closure (89.81%); antimicrobial activity; selective cytotoxicity	254
PGS/gelatin	GO; Clay	Scaffold through *in situ* polymerization	Enhanced thermal and mechanical properties; controlled degradation; homogeneous filler dispersion	147
SA grafted AAc	GO; nHA@SiO_2_	Bone – bioactive porous scaffold	*In vitro* study: enhanced osteoblast proliferation and adhesion; increased mineralization; improved hydrophilicity and porosity	150
ARX and BG	GO; nHA	Bone – porous hydrogel scaffold	High compressive strength and modulus; optimal porosity (∼55%) for cell migration; promoted osteogenic differentiation	255
Poly(acrylic acid) hybrid	GO; nHA; TiO_2_; Ag-sulfadiazine	Bone – antibacterial fracture scaffold	Sustained silver drug release; increased mechanical strength; enhanced osteoblast adhesion and proliferation	151
PPF	GO nanoplatelets, MoS_2_ nanoplatelets	Bone – porous scaffold	Enhanced compressive modulus (up to 108%); high cytocompatibility; ECM deposition	256
PPF	CNTs, GO nanoribbons/nanoplatelets	Bone – tissue scaffold	High cell viability; mild degradation cytotoxicity; enhanced spreading and attachment	257
PCL (3D-printed)	GO (mussel-inspired coating)	Bone – surface-modified scaffold	Improved osteoblast proliferation and differentiation; increased alkaline phosphatase activity and calcium deposition	172
PCL	Graphene/GO	Bone – 3D-printed scaffold	Enhanced modulus (136.74 MPa); cell proliferation; trabecular bone mimicry	242
PLA	GO	Bone – 3D printed scaffold	Improved biocompatibility; 2× mineralization; 30% increase in Young's modulus	173 and 174
GG hydrogel film	rGO; TiO_2_ nanowires	Skin – wound healing hydrogel	*In vitro* study: stimulated fibroblast migration and wound closure; bioactive composite aiding skin regeneration	258

## 6 Future outlook

The integration of biodegradable polymers with graphene-based nanomaterials has produced a formidable class of BGNs with multifunctional advantages for biomedicine. This review has detailed their significant advancements in both controlled drug delivery and tissue engineering. Looking forward, the primary goal is to translate these promising materials from the laboratory to clinical settings. This transition requires comprehensive long-term *in vivo* studies focusing on BGN biocompatibility, degradation kinetics, biodistribution, and immunogenicity. Standardized testing protocols and adherence to good manufacturing practice guidelines are critical hurdles for achieving regulatory approval and enabling scalable, reproducible production.

Future research will likely focus on several key areas. One is the development of smart, multifunctional platforms (theranostics) capable of simultaneous diagnosis and therapy. At the chemical level, this will require more precise tailoring of interfacial chemistry between graphene derivatives and polymers. Another is 4D bioprinting, which can create scaffolds that change their shape or function in response to physiological stimuli after implantation. Advances here will depend not only on printing resolution but also on chemical innovation in photo-crosslinkable or reversible-bonding polymers, which dictate scaffold adaptability and long-term stability. BGNs are also exceptionally well-suited for personalized and AI-driven medicine, where therapies could be tailored based on a patient's specific needs. Mechanistic studies on hydrolytic, enzymatic, and oxidative degradation pathways will be critical to integrate predictive modeling with clinical translation. Finally, a growing focus on bioinspired and sustainable design, using green synthesis methods and mimicking natural tissue structures, will continue to drive the field forward. In particular, greener chemical synthesis routes for graphene and biodegradable polymers can help reduce cytotoxic byproducts while maintaining functional surface chemistry, aligning with both regulatory and sustainability goals.

## 7 Conclusions

BGNs represent a powerful convergence of nanotechnology, materials science, and biomedicine. These materials synergistically combine the exceptional mechanical, electrical, and responsive properties of graphene with the biocompatibility and sustainability of biodegradable polymers. In controlled drug delivery, BGNs enhance drug-loading capacity and enable targeted, stimuli-triggered release, improving therapeutic efficacy while minimizing side effects. In tissue engineering, they function as robust scaffolds that promote cellular growth and guide the regeneration of diverse tissues, including bone, skin, and cartilage. The fabrication of BGNs has progressed from simple solution-based methods to advanced techniques like 3D bioprinting, where innovations in crosslinking chemistry, surface functionalization, and reversible bonding are crucial for scaffold adaptability and stability. While BGNs hold immense promise for sustainable and effective biomedical solutions, further research, particularly rigorous *in vivo* experiments and clinical trials, is essential to advance these innovative materials toward practical medical applications.

## Author contributions

M. Mohiuddin: writing – original draft, writing – review and editing, methodology. M. M. Rahman: writing – original draft, supervision, project administration, formal analysis, conceptualization. M. N. Uddin: writing – review and editing, supervision, project administration, conceptualization. R. Hasan: writing – review and editing, methodology, investigation. I. Rahman: writing – review and editing, investigation, formal analysis, visualization.

## Conflicts of interest

The authors declare that they have no known competing financial interests or personal relationships that could have appeared to influence the work reported in this paper.

## Data Availability

All data used to prepare the manuscript are included. Additional explanations will be available upon request.
